# Supplementary material for: DNA methylation modulates nucleosome retention in sperm and H3K4 methylation deposition in early mouse embryos
Source: Nat Commun. 2025 Jan 7;16:465. doi: 10.1038/s41467-024-55441-1 (PMC11706963; doi:10.1038/s41467-024-55441-1)
Supplement: Supplementary file 1 — Supplementary Information [file 41467_2024_55441_MOESM1_ESM.pdf]

DNA methylation modulates nucleosome retention in sperm and H3K4 methylation deposition in early mouse embryos.

Grigorios Fanourgakis[1], Laura Gaspa-Toneu[1, 2], Pavel A. Komarov[1, 2], Panagiotis Papasaikas[1], Evgeniy A. Ozonov[1], Sebastien A. Smallwood[1] and Antoine H.F.M. Peters[1, 2].*

SUPPLEMENTARY FIGURE LEGENDS

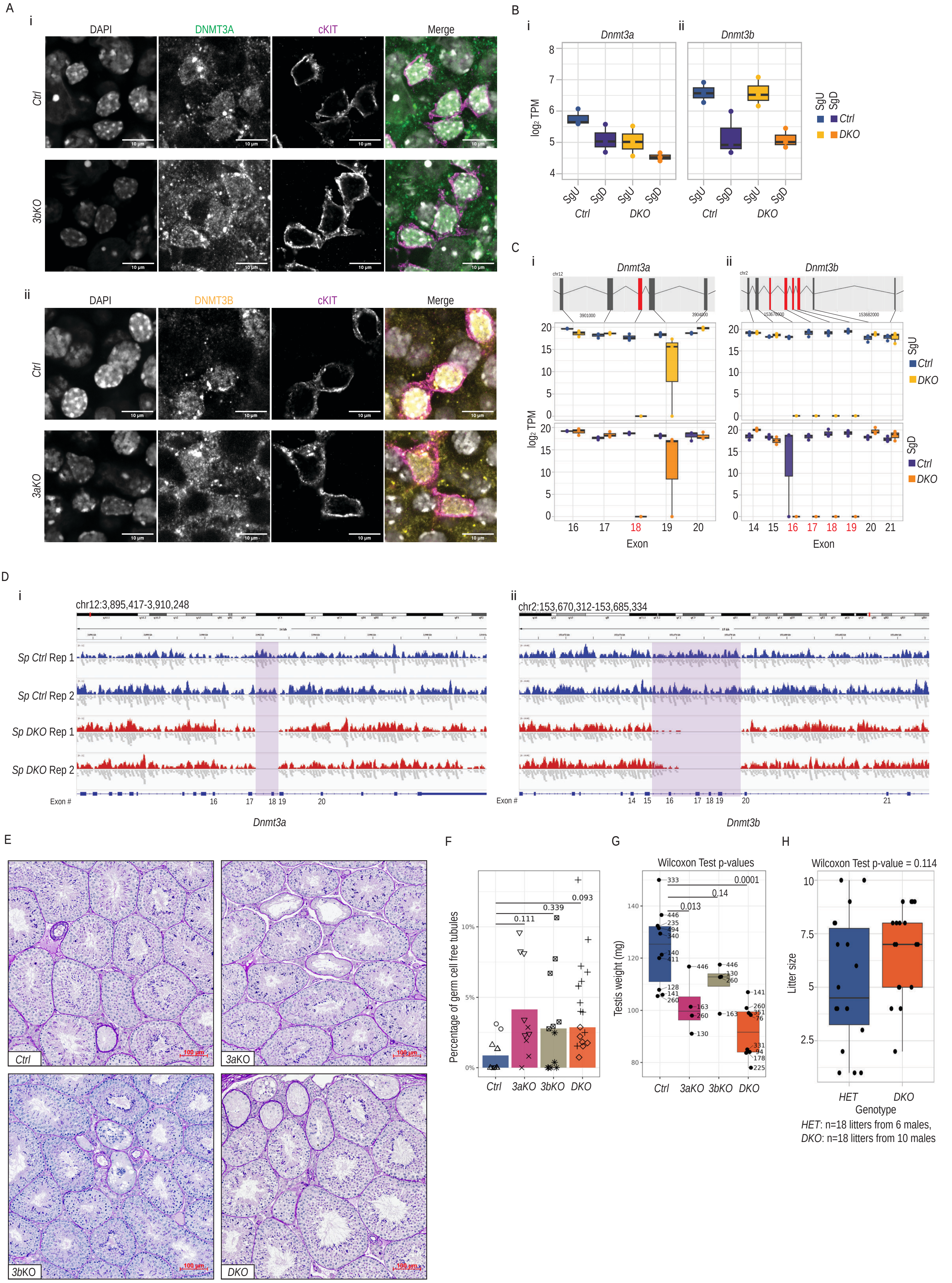
A
i
DAPI
DNMT3A
cKIT
Merge
Ctrl
3bKO
ii
DAPI
DNMT3B
cKIT
Merge
Ctrl
3aKO
B
i
Dnmt3a
ii
Dnmt3b
log2 TPM
SgU
SgD
Ctrl
DKO
C
i
Dnmt3a
ii
Dnmt3b
log2 TPM
Exon
D
i
chr12:3,895,417-3,910,248
Sp Ctrl Rep 1
Sp Ctrl Rep 2
Sp DKO Rep 1
Sp DKO Rep 2
Exon #
Dnmt3a
ii
chr2:153,670,312-153,685,334
Dnmt3b
E
Ctrl
3aKO
3bKO
DKO
F
Percentage of germ cell free tubules
Ctrl
3aKO
3bKO
DKO
G
Wilcoxon Test p-values
Testis weight (mg)
H
Wilcoxon Test p-value = 0.114
Litter size
HET
DKO
Genotype
HET: n=18 litters from 6 males,
DKO: n=18 litters from 10 males

Supplementary Figure 1. Characterization of *Dnmt3a* and/or *Dnmt3b* conditional depletion in male germ cells.

A) Whole mount immunofluorescence staining of (i) DNMT3A in seminiferous tubules of *Ctrl* (n=1) and *3bKO* (n=1) testes, and (ii) DNMT3B in seminiferous tubules of *Ctrl* (n=1) and *3aKO* (n=1) testes. Samples were co-stained with cKIT and DNA was visualized by DAPI. Maximum projections of multiple confocal z-stacks are shown. Scale bars = 10 μm.

B) Boxplots showing log_2TPM values for the (i) *Dnmt3a* and (ii) *Dnmt3b* genes in undifferentiated spermatogonia (SgU) and differentiated spermatogonia (SgD) from *Ctrl* and *DKO* samples (n=3 for each biological sample type).

C) Boxplots showing the log_2TPM values for the floxed exons (in red) and their surrounding exons (in gray) of (i) *Dnmt3a* and (ii) *Dnmt3b* genes in SgU and SgD from *Ctrl* and *DKO* samples (n=3 for each biological sample type).

D) IGV genome browser view of aligned whole genome EM-seq reads across the (i) *Dnmt3a* and (ii) *Dnmt3b* genes from 2 replicates of *Ctrl* and 2 replicates of *DKO* sperm samples. The genomic loci flanked by loxP sites are highlighted in purple.

E) Representative staining with periodic acid-Schiff's and hematoxylin of stage IV-V seminiferous tubule sections from *Ctrl*, *3aKO*, *3bKO* and *DKO* testes. Scale bars = 50 μm.

F) Barplot representing the summed percentage of germ cell free seminiferous tubules for each genotype group, while points show individual section percentages for each animal, distinguished by different shapes for *Ctrl* (n=2 animals, n=12 sections, n= 3210 seminiferous tubules), *3aKO* (n=2 animals, n=10 sections, n= 2558 seminiferous tubules), *3bKO* (n=2 animals, n=12 sections, n= 2975 seminiferous tubules) and *DKO* (n=3 animals, n=10 sections, n= 2295 seminiferous tubules) animals. Statistical comparisons were performed using a mixed-effects logistic regression model, accounting for both biological and technical replicates.

G) Boxplots showing testicular weight from *Ctrl* (n=10), *3aKO* (n=4), *3bKO* (n=4) and *DKO* (n=8) animals. Each dot represents one animal with the corresponding age reported. P-values between biological groups were calculated by two-sided Wilcoxon Test.

H) Boxplots showing number of pups per litter sired either from Het (n=18 litters from 6 sires) and *DKO* (n=18 litters from 10 sires) animals. Each dot represents a litter. P-values between biological groups were calculated by two-sided Wilcoxon Test. The boxes extend from the first quartile (Q1) to the third quartile (Q3) of the data distribution, with a line in the box representing the median. Whiskers indicate variability outside Q1 and Q3, extending to the minimum and maximum values within 1.5 times the interquartile range (IQR).

Fanourgakis et al Supplementary Figure 2

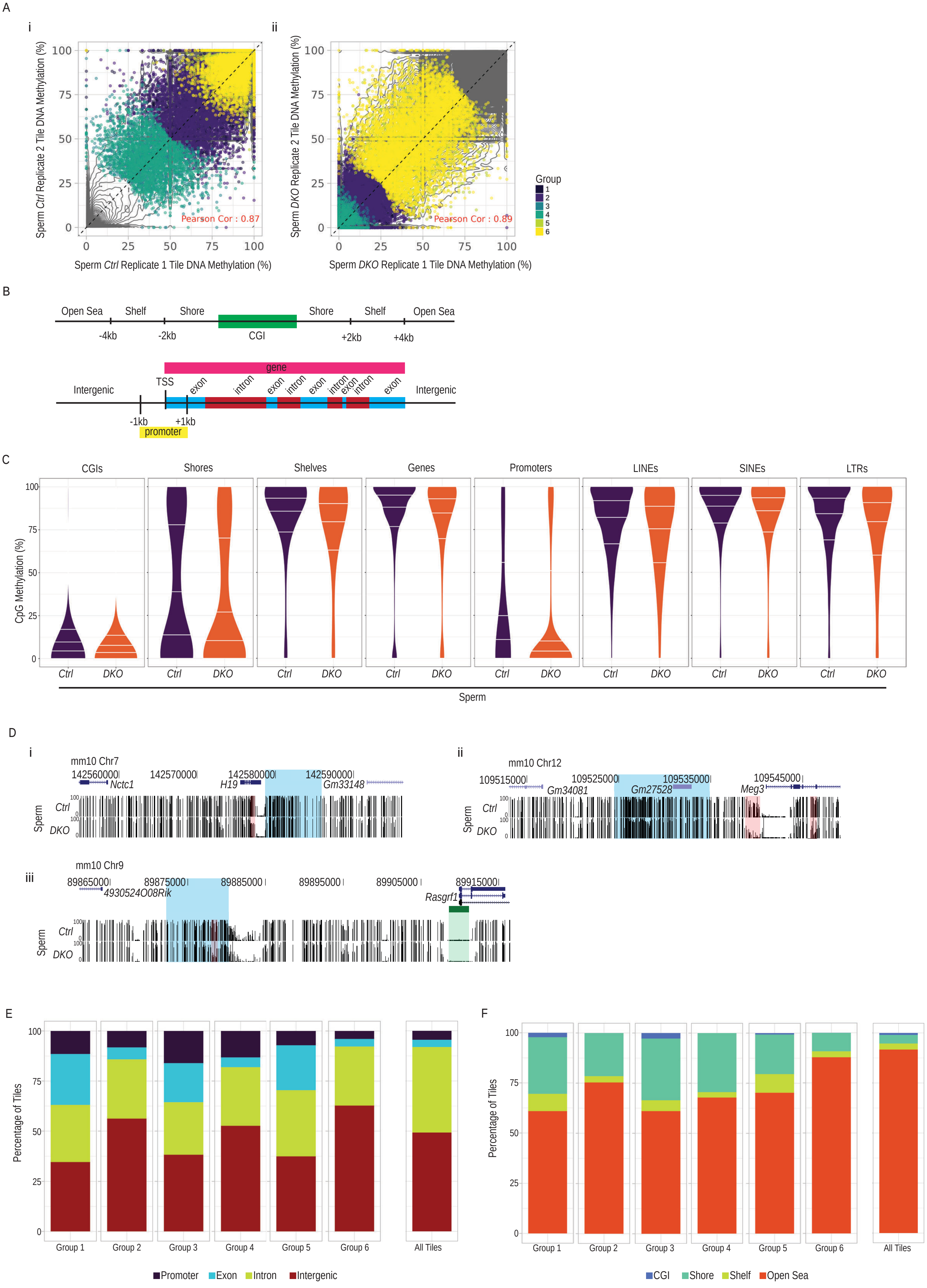

Supplementary Figure 2. DNAme of various genomic features and paternal gametic DMRs in *DKO* sperm.

A) Scatterplot showing percentage DNAme of 500bp genomic tiles assessed by EMseq in (i) replicate 1 *Ctrl* (x-axis) and replicate 2 *Ctrl* (y-axis) sperm and (ii) replicate 1 *DKO* (x-axis) and replicate 2 *DKO* (y-axis) sperm. HypoDMR tiles are highlighted according to the group that they belong according to Figure 1E. Contour lines denote the density of NonDMR tiles. The Pearson correlation coefficient between rep1 and rep2 for each genotype is presented in lower right corner.

B) Schematic representation of genomic feature annotation related to CpG islands (top) and genes (bottom).

C) Violin plots showing the percentage of DNAme of genomic CpGs that overlap with CGI, gene and repeat related genomic features in *Ctrl* (n=2) and *DKO* (n=2) sperm. The center lines in the violins represent median values. The upper and lower lines indicate the interquartile range (IQR; from the 25th to 75th percentile).

D) UCSC genome browser snapshot of the (i) H19/Igf2, (ii) Dlk1-Gtl2 and (iii) Rasgrf1 paternally methylated gametic DMRs, showing tracks for DNAme levels in *Ctrl* and *DKO* sperm. Gametic DMRs are highlighted in blue. The promoter CGI and the hypoDMR are highlighted in green and red respectively.

E) Bar plot showing the percentage of hypoDMRs in each group as defined in Figure 1E overlapping with gene related features. As a reference, the percentage of gene related features at all genomic tiles is shown on the right.

F) Bar plot showing the percentage of hypoDMRs in each group as defined in Figure 1E overlapping with CpG island related features. As a reference, the percentage of CpG island related features at all genomic tiles is shown on the right.

Fanourgakis et al Supplementary Figure 3

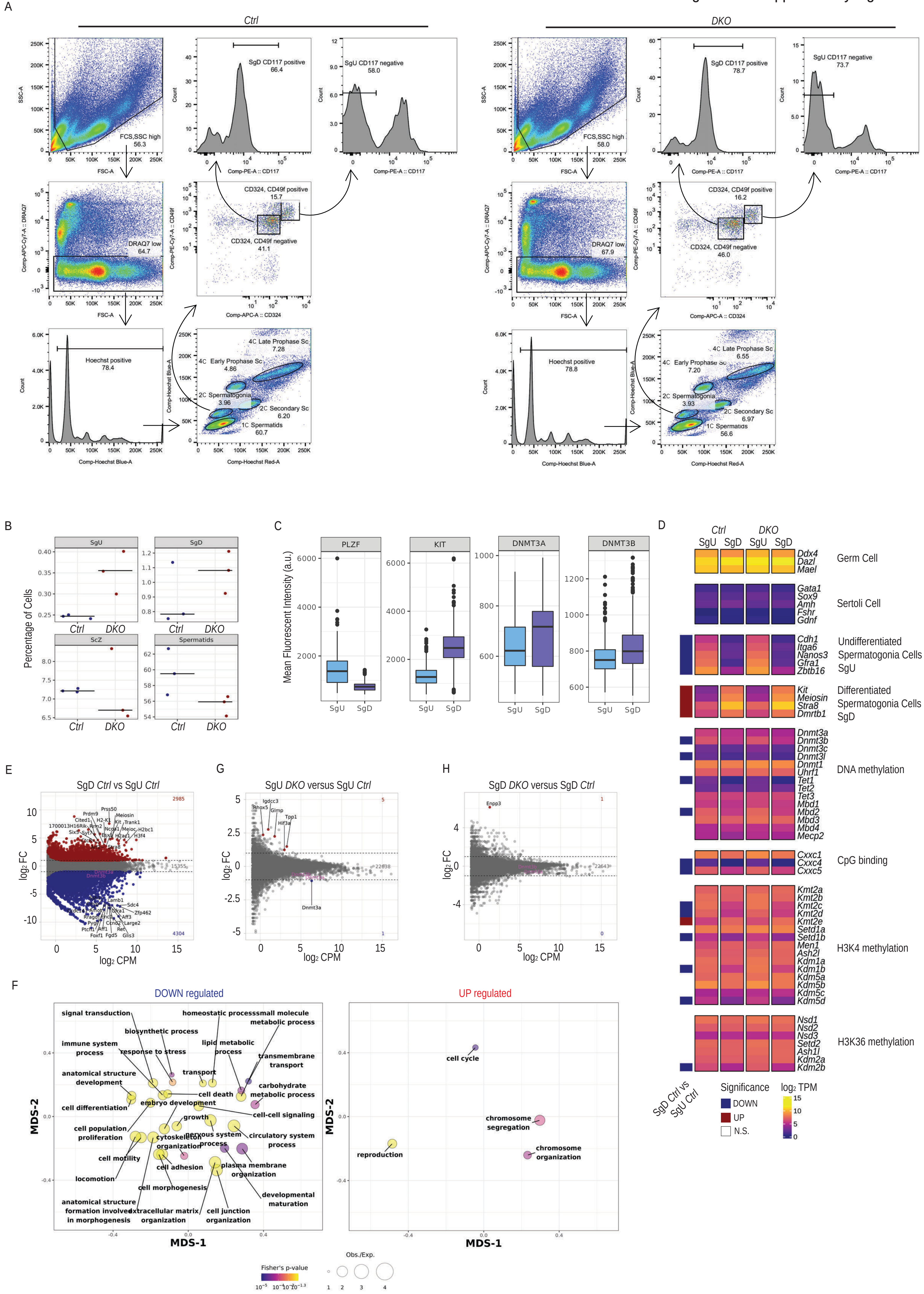

Supplementary Figure 3. Transcriptional effects of *Dnmt3a*/*Dnmt3b* depletion in spermatogonia.

A) Representative FACS sorting profiles of germ cells with gating parameters from adult *Ctrl* (left) and *DKO* (right) male mice. FSC versus SSC plot indicates gate excluding debris. FCS versus DRAQ7 plot indicates gate including viable cells. Hoechst-Blue histogram indicates gate with stained cells. Hoechst-Red versus Hoechst-Blue plot indicates gates containing populations of spermatogonia, meiotic spermatocytes and post-meiotic cells. CD324 (E-CADHERIN) versus CD49f (INTEGRIN ALPHA 6) plot showing gates containing undifferentiated spermatogonia SgU (CD324^{high}, CD49f^{high}) and differentiated spermatogonia SgD (CD324^{low}, CD49f^{low}). CD117 (c-KIT) histogram indicates the gate with SgU spermatogonia (Hoechst-Bluemid, Hoechst-Redlow, CD324^{high}, CD49f^{high}, CD117^{low}). CD117 (c-KIT) histogram indicates the gate with SgD spermatogonia (Hoechst-Blue^{mid}, Hoechst-Red^{low}, CD324^{low}, CD49f^{low}, CD117^{high}).

B) Dotplots representing the relative percentages of sorted SgU, SgD, Zygotene Spermatocytes (ScZ) and spermatids cells to all viable Hoechst positive cells from *Ctrl* (n=3) and *DKO* (n=3) animals.

C) Boxplots showing from left to right the mean fluorescent intensity value of a-PLZF, a-cKIT, a-DNMT3A and a-DNMT3B immunofluorescence staining of nuclei from FACS sorted SgU and SgD from *Ctrl* animals.

D) Heatmap showing the $\log_2$TPM values of selected gene markers in SgU and SgD from *Ctrl* (n=3) and *DKO* (n=3) samples.

E) Scatterplots showing gene expression $\log_2$ fold changes among *Ctrl* SgD (n=3) versus *Ctrl* SgU (n=3) samples were plotted against $\log_2$CPM gene expression levels. The numbers of significantly upregulated (red) or downregulated (blue) genes are displayed on the left and genes names of the most highly significant differentially expressed genes are shown.

F) Bubbleplots showing significantly enriched GO-terms associated with downregulated (left) and upregulated (right) genes in *Ctrl* SgD versus *Ctrl* SgU samples. The size of the bubble denotes enrichment and color denotes significance. Distance of the bubbles centers represents similarity of genes contained in each GO-term.

G) Scatterplots showing gene expression $\log_2$ fold changes among *DKO* SgU (n=3) versus *Ctrl* SgU (n=3) samples were plotted against $\log_2$CPM gene expression levels. The numbers of significantly upregulated (red) or downregulated (blue) genes are displayed on the right.

H) Scatterplots showing gene expression $\log_2$ fold changes among *DKO* SgD (n=3) versus *Ctrl* SgD (n=3) samples were plotted against $\log_2$CPM gene expression levels. The numbers of significantly upregulated (red) or downregulated (blue) genes are displayed on the right.

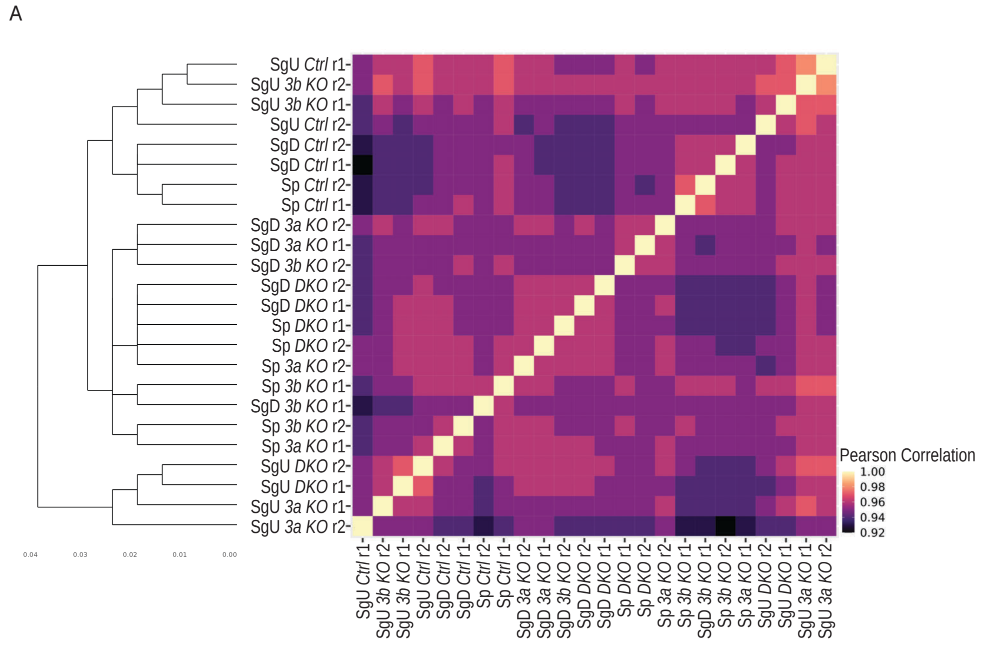

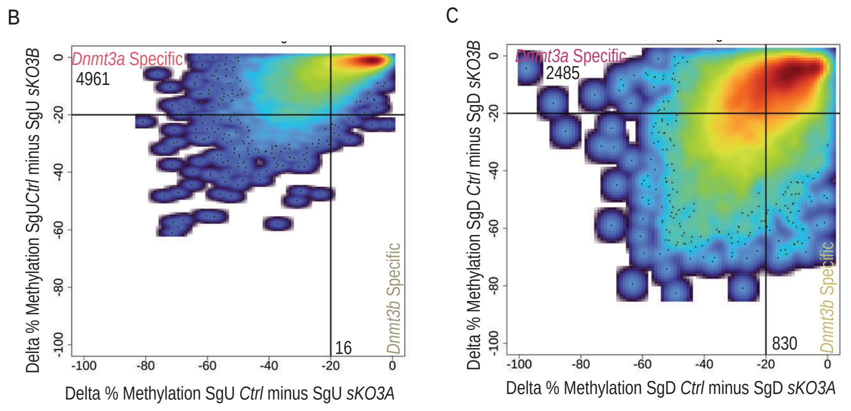

D

	SgU	SgD	Sp
Rest	203695	208696	208142
DKO specific	6126	2787	2906
Dnmt3A specific	4961	2485	3449
Dnmt3B specific	16	830	301

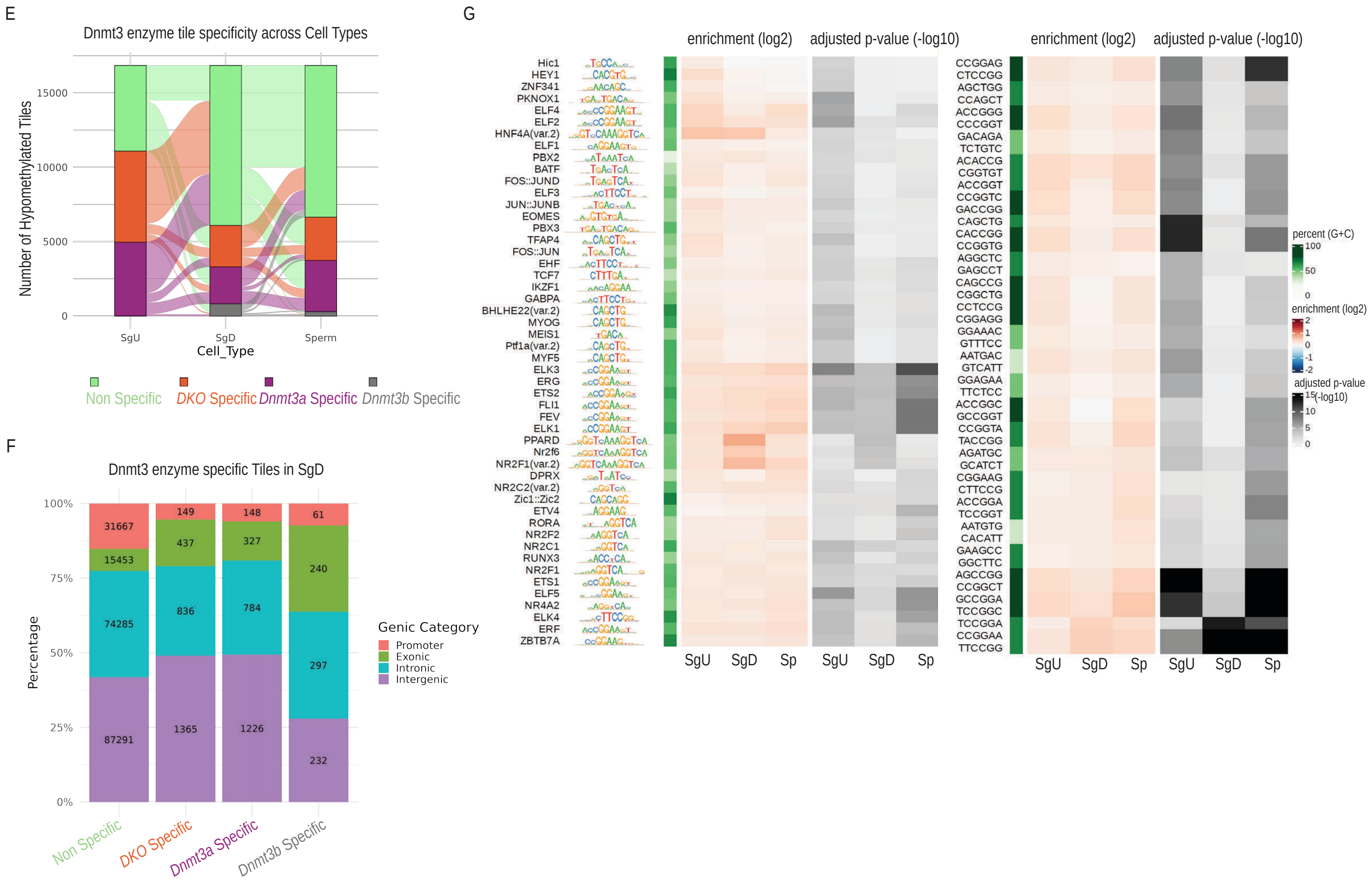

Supplementary Figure 4. DNAme levels of hypoDMRs during spermatogonia development upon *Dnmt3a* and/or *Dnmt3b* depletion.

A) Heatmap showing the Pearson correlation coefficient of DNAme percentage at all genomic tiles detected by RRBS between the biological replicates of undifferentiated spermatogonia (SgU), differentiated spermatogonia (SgD) and sperm from *Ctrl*, *DKO*, *3aKO* and *3bKO* samples.

B) Scatterplots showing DNAme difference between *Ctrl* SgU minus *3aKO* SgU (x-axis) versus DNAme difference between *Ctrl* SgU minus *3bKO* SgU (y-axis) for all detected hypomethylated tiles by RRBS. Black lines indicate DNA methylation loss thresholds (20%) to define *Dnmt3a*- and *Dnmt3b*-specfic hypomethylated tiles in SgU.

C) Scatterplots showing DNAme difference between *Ctrl* SgD minus *3aKO* SgD (x-axis) versus DNAme difference between *Ctrl* SgD minus *3bKO* SgD (y-axis) for all detected hypomethylated tiles by RRBS. Black lines indicate DNA methylation loss thresholds (20%) to define *Dnmt3a*- and *Dnmt3b*-specfic hypomethylated tiles in SgD.

D) Table with number of *DKO*-, Dnmt*3a*- and *Dnmt3b*-specfic hypomethylated tiles in different cell types.

E) Alluvial plot showing the changes in DNMT3 enzyme specificity of hypomethylated tiles over different cell types.

F) Bar plot showing the percentage of *DKO*-, *Dnmt3a*- and *Dnmt3b*-specfic hypomethylated tiles in SgD overlapping with gene related features. As a reference, the percentage of gene related features at non-specific tiles is shown on the right.

G) Heatmaps showing $\log_2$ transcription factor (TF) motif (left) or hexanucleotide (right) enrichments and adjusted enrichment p-values for the genomic regions that specifically display methylation loss in DNMT3a KO context in the different stages. Differential enrichment is assessed over the complete set of regions that display similar levels of methylation loss (see also Material and Methods). For every TF its binding motif sequence logo and percent GC context are also shown. All adjusted enrichment p-values are calculated using the Benjamini & Hochberg correction method.

Fanourgakis et al Supplementary Figure 5

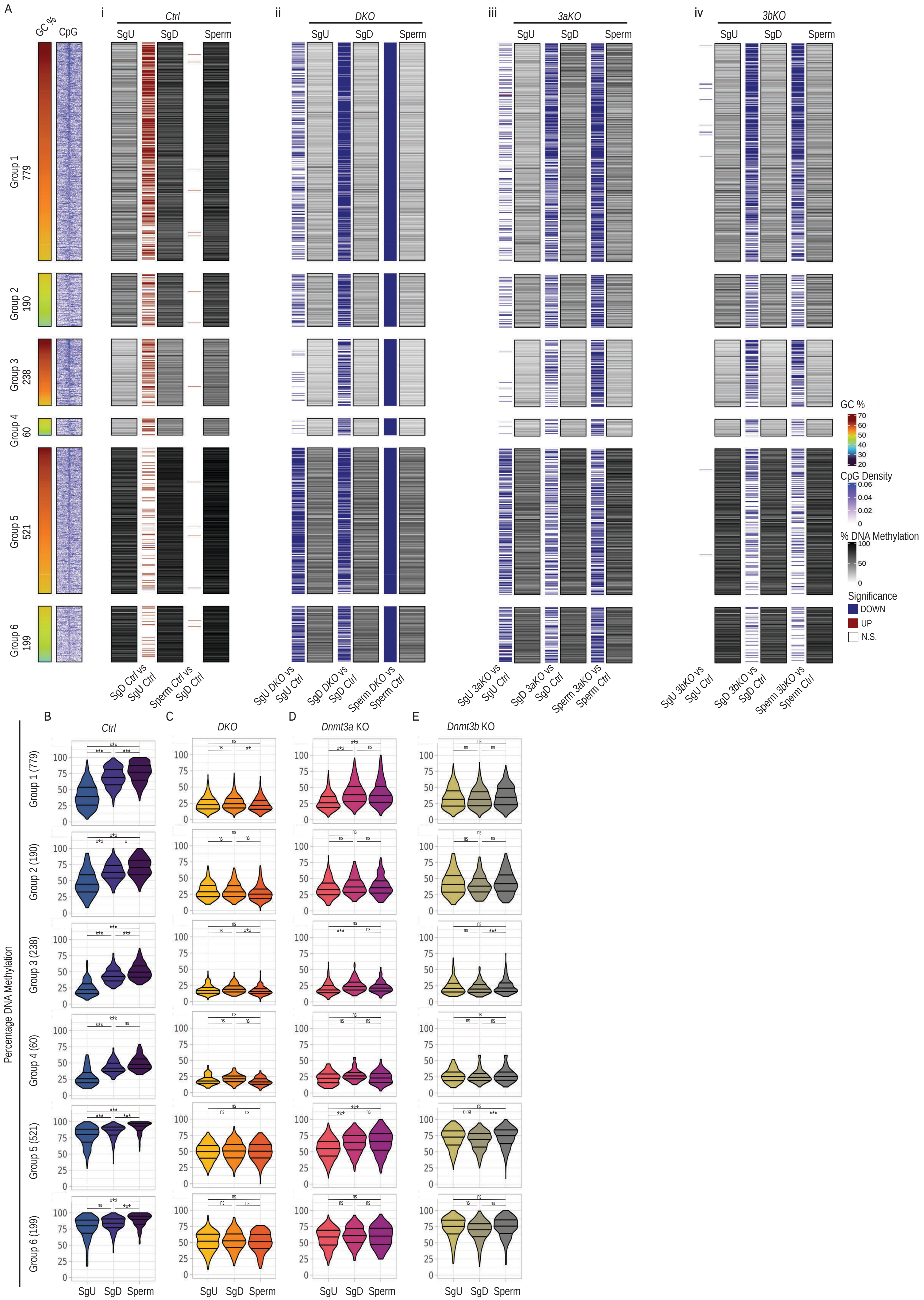

Supplementary Figure 5. Comparison of DNAme levels of hypoDMRs during spermatogonia development upon *Dnmt3a* and/or *Dnmt3b* depletion.

A) Heatmap showing the DNAme percentage of hypoDMRs detected by RRBS in undifferentiated spermatogonia (SgU), differentiated spermatogonia (SgD) and sperm from (i) *Ctrl*, (ii) *DKO*, (iii) *3aKO* and (iv) *3bKO* samples. Grouping and ordering of hypoDMRs is based on Figure 1E. Dark to bright color scale denotes high to low DNAme percentage. Color bands between the DNAme heatmaps denote hypoDMRs with significantly differential DNAme in the contrasts mentioned below them. The density of CpGs at hypoDMRs (±5 kb from their center) is indicated.

B-E) Violin plots showing percentages DNAme of hypoDMRs assessed by RRBS in undifferentiated spermatogonia (SgU), differentiated spermatogonia (SgD) and sperm from (A) *Ctrl* (n=2 from each celltype), (B) *DKO* (n=2 from each celltype), (C) *Dnmt3aKO* (n=2 from each celltype) and (D) *Dnmt3bKO* (n=2 from each celltype) samples. Two-sample, two-sided Wilcoxon tests were performed between indicated groups ns $P>0.05$, * $P < 0.05$, ** $P \leq 0.01$, *** $P \leq 0.001$. Center lines in violin plots represent median values while upper and lower lines indicate the interquartile range (IQR; from the 25th to 75th percentile).

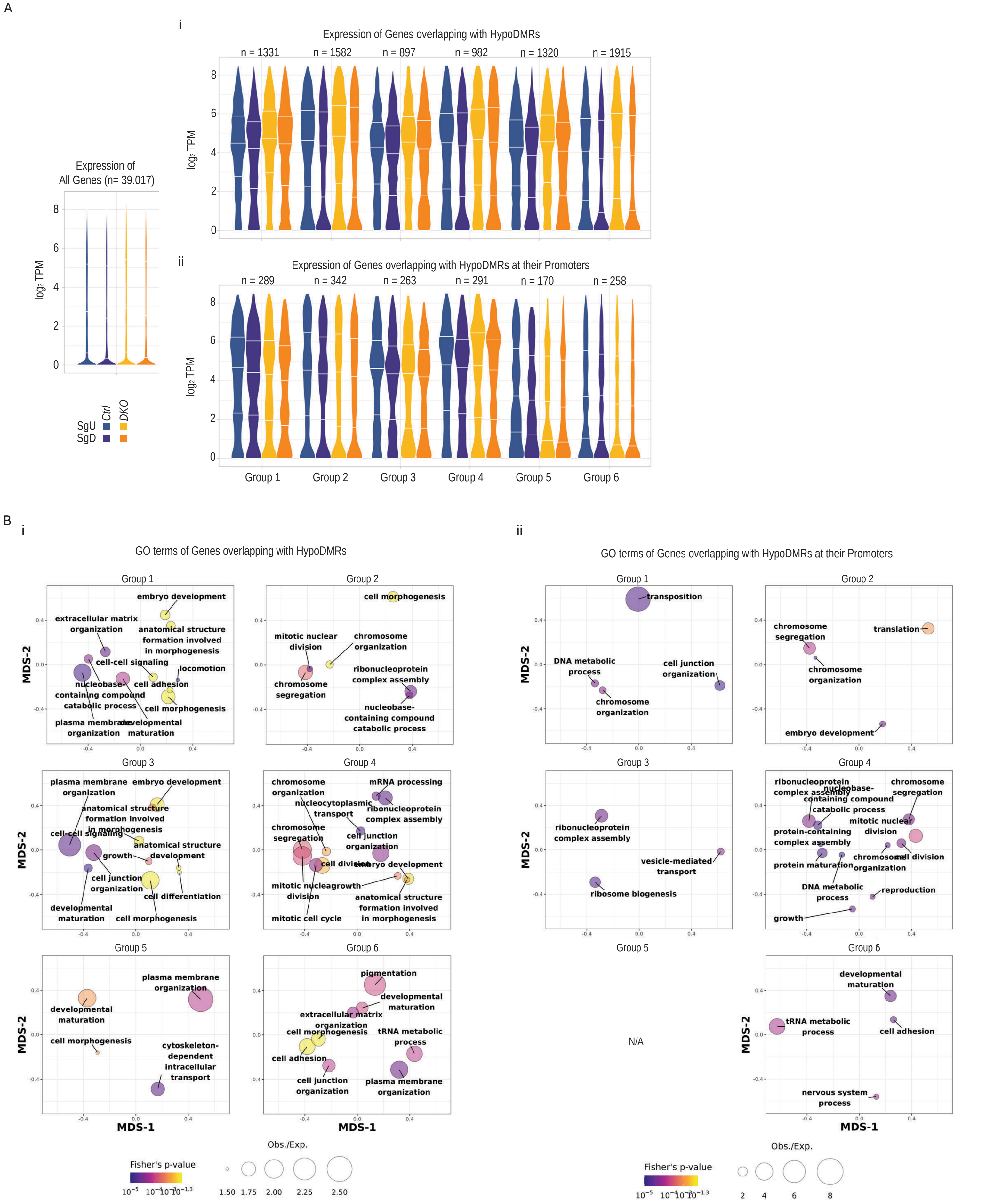
A
i
Expression of Genes overlapping with HypoDMRs
n = 1331
n = 1582
n = 897
n = 982
n = 1320
n = 1915
log2 TPM
Expression of
All Genes (n= 39.017)
log2 TPM
ii
Expression of Genes overlapping with HypoDMRs at their Promoters
n = 289
n = 342
n = 263
n = 291
n = 170
n = 258
log2 TPM
Ctrl
DKO
SgU
SgD
Group 1
Group 2
Group 3
Group 4
Group 5
Group 6
B
i
GO terms of Genes overlapping with HypoDMRs
Group 1
embryo development
extracellular matrix organization
anatomical structure formation involved in morphogenesis
cell-cell signaling
locomotion
nucleobase-containing compound catabolic process
cell adhesion
cell morphogenesis
plasma membrane organization
developmental maturation
Group 2
cell morphogenesis
mitotic nuclear division
chromosome organization
ribonucleoprotein complex assembly
chromosome segregation
nucleobase-containing compound catabolic process
Group 3
plasma membrane organization
embryo development
anatomical structure formation involved in morphogenesis
cell-cell signaling
anatomical structure development
growth
cell junction organization
cell differentiation
developmental maturation
cell morphogenesis
Group 4
chromosome organization
mRNA processing
nucleocytoplasmic transport
ribonucleoprotein complex assembly
chromosome segregation
cell junction organization
cell division
embryo development
mitotic nuclear division
growth
anatomical structure formation involved in morphogenesis
mitotic cell cycle
Group 5
plasma membrane organization
developmental maturation
cell morphogenesis
cytoskeleton-dependent intracellular transport
Group 6
pigmentation
developmental maturation
extracellular matrix organization
cell morphogenesis
tRNA metabolic process
cell adhesion
cell junction organization
plasma membrane organization
MDS-1
MDS-2
Fisher's p-value
Obs./Exp.
1.50
1.75
2.00
2.25
2.50
ii
GO terms of Genes overlapping with HypoDMRs at their Promoters
Group 1
transposition
DNA metabolic process
cell junction organization
chromosome organization
Group 2
chromosome segregation
translation
chromosome organization
embryo development
Group 3
ribonucleoprotein complex assembly
vesicle-mediated transport
ribosome biogenesis
Group 4
ribonucleoprotein complex assembly
nucleobase-containing compound catabolic process
chromosome segregation
mitotic nuclear division
protein-containing complex assembly
protein maturation
chromosome organization
cell division
DNA metabolic process
reproduction
growth
Group 5
N/A
Group 6
developmental maturation
tRNA metabolic process
cell adhesion
nervous system process
MDS-1
MDS-2
Fisher's p-value
Obs./Exp.
2
4
6
8

Supplementary Figure 6. Transcriptional expression and gene ontology of hypoDMRs associated genes.

A) Violin plot showing log_2TPM values of (i) genes overlapping with hypoDMRs grouped according to Figure 1E and (ii) genes overlapping with promoter associated hypoDMRs grouped according to Figure 1E in *Ctrl* SgU (n=3), *Ctrl* SgD (n=3), *DKO* SgU (n=3) and *DKO* SgD (n=3). For reference log_2TPM values of all genes are shown in the left. Center lines in violin plots represent median values while upper and lower lines indicate the interquartile range (IQR; from the 25th to 75th percentile).

B) Bubbleplots showing significantly enriched GO-terms associated with (i) genes overlapping with hypoDMRs and (ii) genes overlapping with promoter associated hypoDMRs. The size of the bubble denotes enrichment and color denotes significance. Distance of the bubbles centers represents that similarity of genes contained in each GO-term.

Fanourgakis et al Supplementary Figure 7

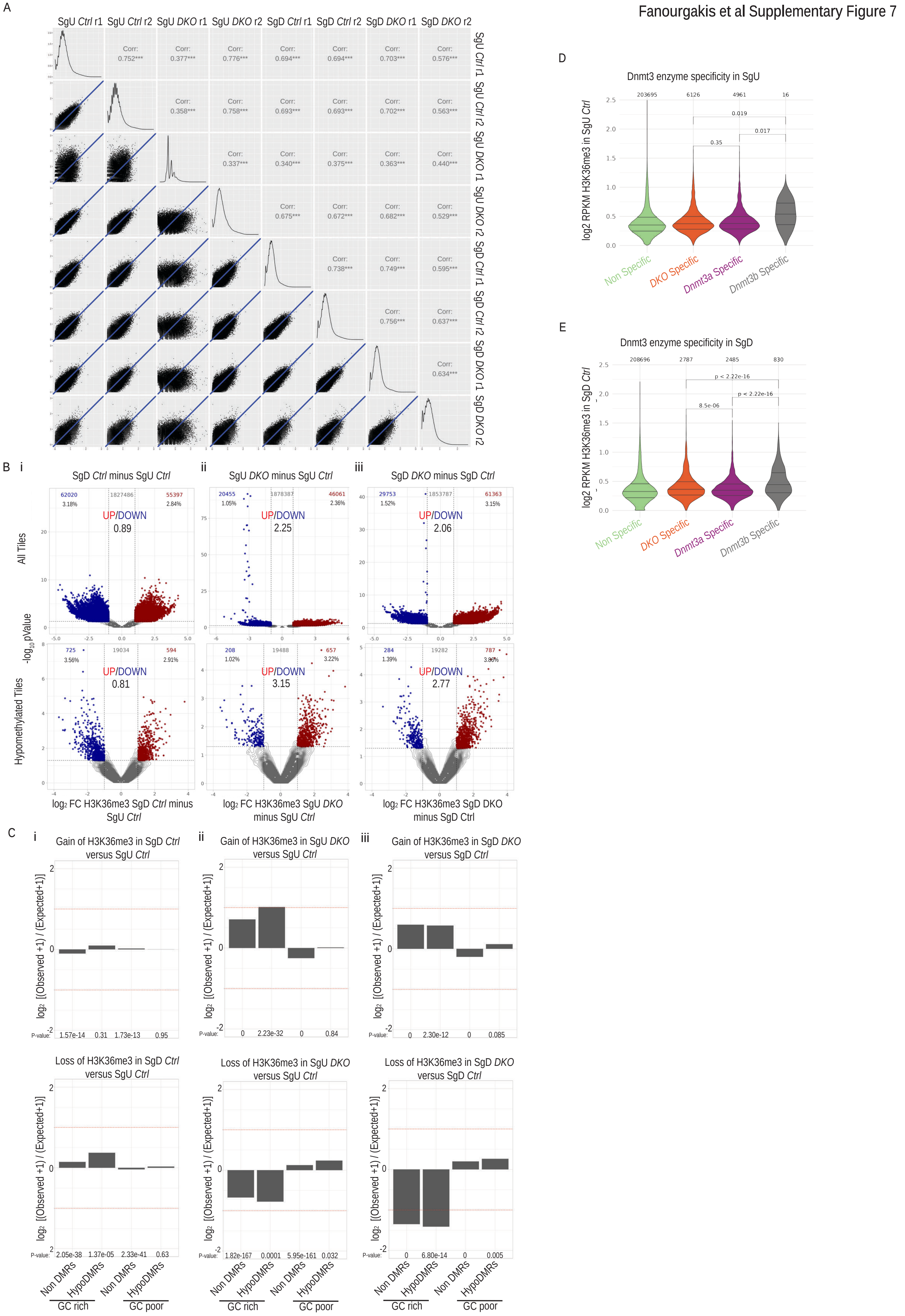

Supplementary Figure 7. Differential H3K36me3 analysis in *DKO* spermatogonia.

A) Scatterplots [left side] show the H3K36me3 log_2RPKM values at 100.000 randomly selected genomic tiles detected by ULI-NChIP-seq between pairs of biological replicated samples of undifferentiated spermatogonia (SgU), differentiated spermatogonia (SgD) from *Ctrl* and *DKO* animals. The diagonal line plots show the distribution of the H3K36me3 log_2RPKM values in each replicate. The Pearson correlation coefficient [right side] of H3K36me3 log_2RPKM values at 100.000 randomly selected genomic tiles detected by ULI-NChIP-seq between the biological replicates of undifferentiated spermatogonia (SgU), differentiated spermatogonia (SgD) from *Ctrl* and *DKO* samples.

B) Volcano plots showing differential enrichment for H3K36me3 in (i) *Ctrl* SgD (n=2) versus *Ctrl* SgU (n=2), (ii) *DKO* SgU (n=2) versus *Ctrl* SgU (n=2) and (iii) *DKO* SgD (n=2) versus *Ctrl* SgD (n=2) (right) contrasts for all genomic tiles (upper) and hypoDMR tiles (lower). Tiles with statistically significant gain and loss of the H3K36me3 signals are highlighted in red (upregulated) and blue (downregulated) and their numbers and percentage of differentially are shown on the upper right and left corners respectively. The ratio between upregulated versus downregulated tiles in each plot is shown in the middle.

C) Bar plots showing log_2 enrichments of GC-rich or GC-poor nonDMR and hypoDMR tiles among tiles with significant H3K36me3 gain (upper) or significant H3K36me3 loss (lower) in (i) *Ctrl* SgD versus *Ctrl* SgU, (ii) *DKO* SgU versus *Ctrl* SgU and (iii) *DKO* SgD versus *Ctrl* SgD. P-values were calculated by Fisher's exact test.

D) Violin plots showing the log_2RPKM H3K36me3 in *Ctrl* SgU of none-specific tiles, DKO-, *Dnmt3a*- and *Dnmt3b*-specfic hypomethylated tiles in SgU. The center lines in the violins represent median values. The upper and lower lines indicate the interquartile range (IQR; from the 25th to 75th percentile). P-values between were calculated by two-sided Wilcoxon Test.

E) Violin plots showing the log_2RPKM H3K36me3 in *Ctrl* SgD of none-specific tiles, DKO-, *Dnmt3a*- and *Dnmt3b*-specfic hypomethylated tiles in SgD. The center lines in the violins represent median values. The upper and lower lines indicate the interquartile range (IQR; from the 25th to 75th percentile). P-values between were calculated by two-sided Wilcoxon Test.

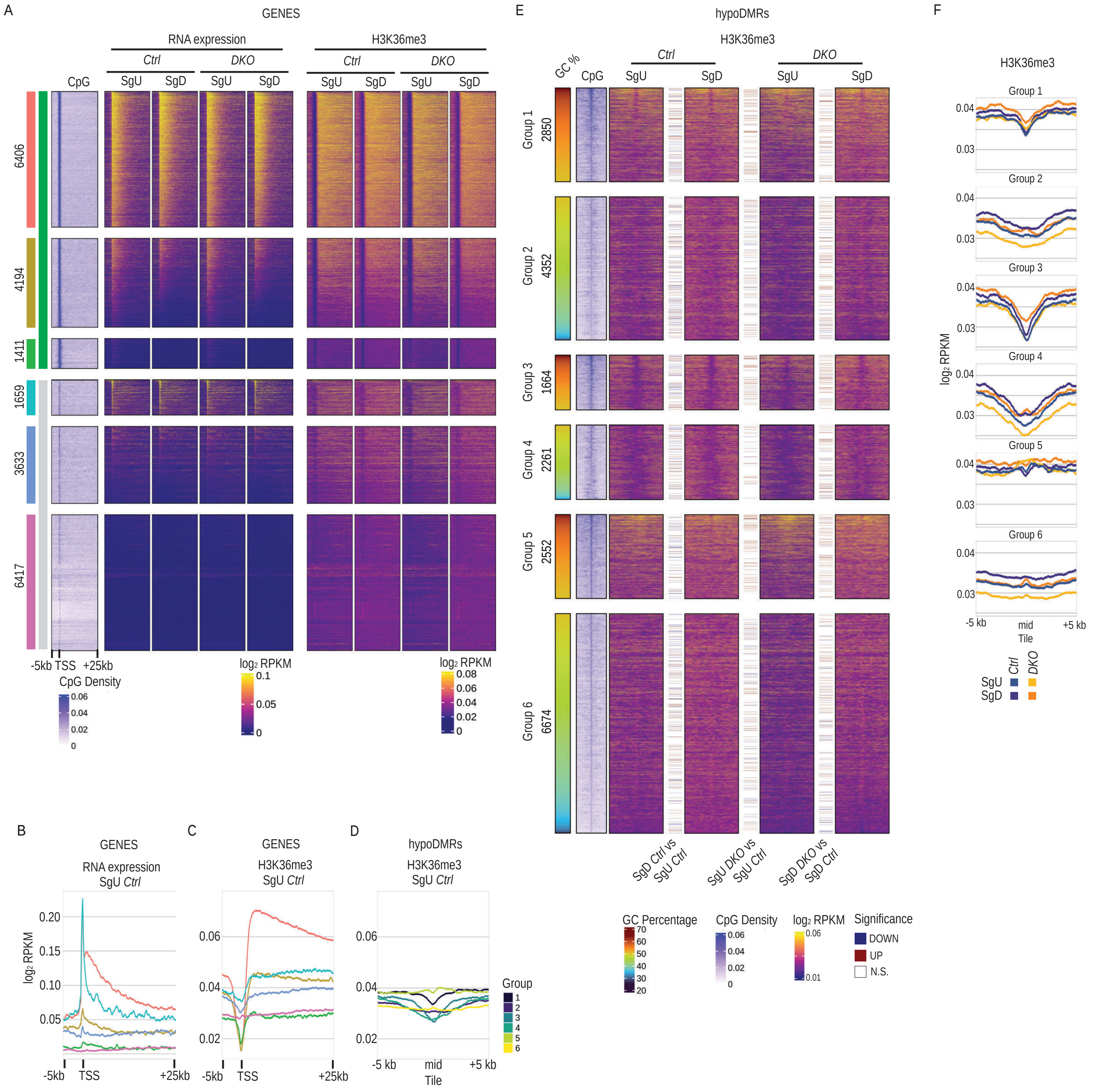
A
GENES
RNA expression
H3K36me3
Ctrl
DKO
CpG
SgU
SgD
6406
4194
1411
1659
3633
6417
-5kb TSS +25kb
CpG Density
0.06
0.04
0.02
0
log2 RPKM
0.1
0.05
0
log2 RPKM
0.08
0.06
0.04
0.02
0
B
GENES
RNA expression
SgU Ctrl
log2 RPKM
0.20
0.15
0.10
0.05
-5kb
TSS
+25kb
C
GENES
H3K36me3
SgU Ctrl
0.06
0.04
0.02
-5kb
TSS
+25kb
D
hypoDMRs
H3K36me3
SgU Ctrl
0.06
0.04
0.02
-5 kb
mid
+5 kb
Tile
Group
1
2
3
4
5
6
E
hypoDMRs
H3K36me3
GC %
CpG
Ctrl
DKO
SgU
SgD
Group 1 2850
Group 2 4352
Group 3 1664
Group 4 2261
Group 5 2552
Group 6 6674
SgD Ctrl vs SgU Ctrl
SgU DKO vs SgU Ctrl
SgD DKO vs SgD Ctrl
GC Percentage
70
60
50
40
30
20
CpG Density
0.06
0.04
0.02
0
log2 RPKM
0.06
0.01
Significance
DOWN
UP
N.S.
F
H3K36me3
log2 RPKM
Group 1
Group 2
Group 3
Group 4
Group 5
Group 6
0.04
0.03
-5 kb
mid
+5 kb
Tile
Ctrl
DKO
SgU
SgD

Supplementary Figure 8. H3K36me3 levels of hypoDMRs in *DKO* spermatogonia.

A) Heatmaps showing RNA and H3K36me3 occupancy at gene transcriptional start sites (TSS) (-5/+25kb) in *Ctrl* and *DKO* SgU and SgD (n=2 for each sample). The CpG density at gene TSS (-5/+25kb) is indicated. Genes have been grouped according to the presence of a CpG island at their TSS +/- 1kb and ordered by decreasing expression in *Ctrl* SgU.

B) Line plots showing average RNA occupancy at gene TSS (-5/+25kb) in *Ctrl* SgU (n=2) for each expression group.

C) Line plots showing average H3K36me3 occupancy at gene TSS (-5/+25kb) in *Ctrl* SgU (n=2) for each expression group.

D) Line plots showing average H3K36me3 occupancy at Groups 1-6 hypoDMRs (±5kb) in *Ctrl* SgU (n=2).

E) Heatmaps showing H3K36me3 occupancy at center of hypoDMRs (±5kb) belonging to Groups 1-6 in *Ctrl* and *DKO* SgU and SgD (n=2 for each sample). Colored lines between heatmaps denote hypoDMRs with significantly differential H3K36me3 occupancy in the indicated contrasts. The GC percentage and CpG density at hypoDMRs (±5 kb) are indicated.

F) Line plots showing average H3K36me3 occupancy at Groups 1-6 hypoDMRs (±5kb) in *Ctrl* and *DKO* SgU and SgD (n=2 for each sample).

A

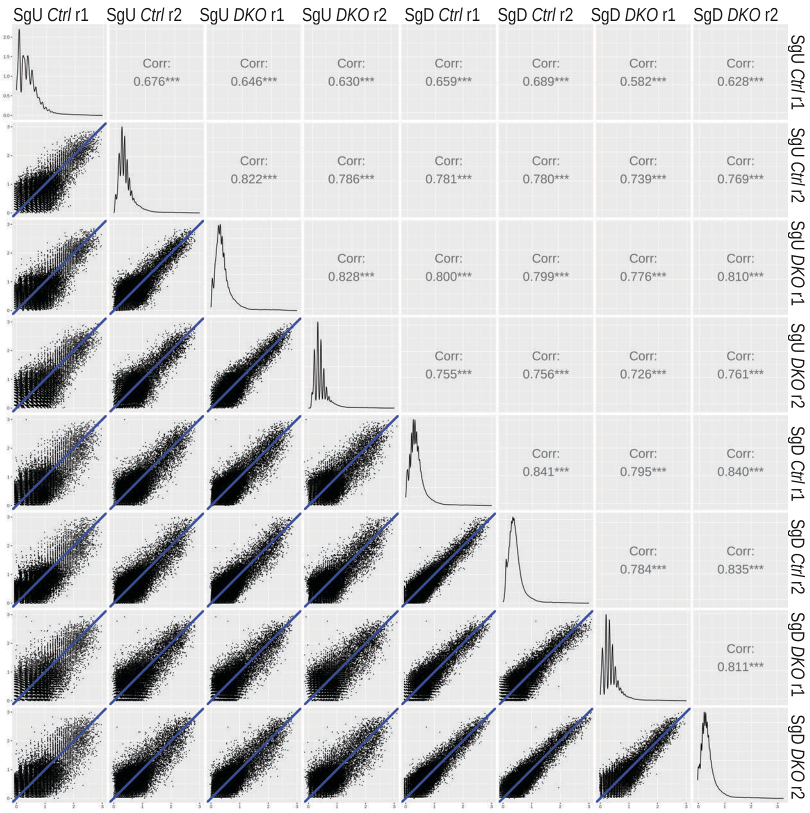
SgU Ctrl r1
SgU Ctrl r2
SgU DKO r1
SgU DKO r2
SgD Ctrl r1
SgD Ctrl r2
SgD DKO r1
SgD DKO r2
Corr: 0.676***
Corr: 0.646***
Corr: 0.630***
Corr: 0.659***
Corr: 0.689***
Corr: 0.582***
Corr: 0.628***
Corr: 0.822***
Corr: 0.786***
Corr: 0.781***
Corr: 0.780***
Corr: 0.739***
Corr: 0.769***
Corr: 0.828***
Corr: 0.800***
Corr: 0.799***
Corr: 0.776***
Corr: 0.810***
Corr: 0.755***
Corr: 0.756***
Corr: 0.726***
Corr: 0.761***
Corr: 0.841***
Corr: 0.795***
Corr: 0.840***
Corr: 0.784***
Corr: 0.835***
Corr: 0.811***

B

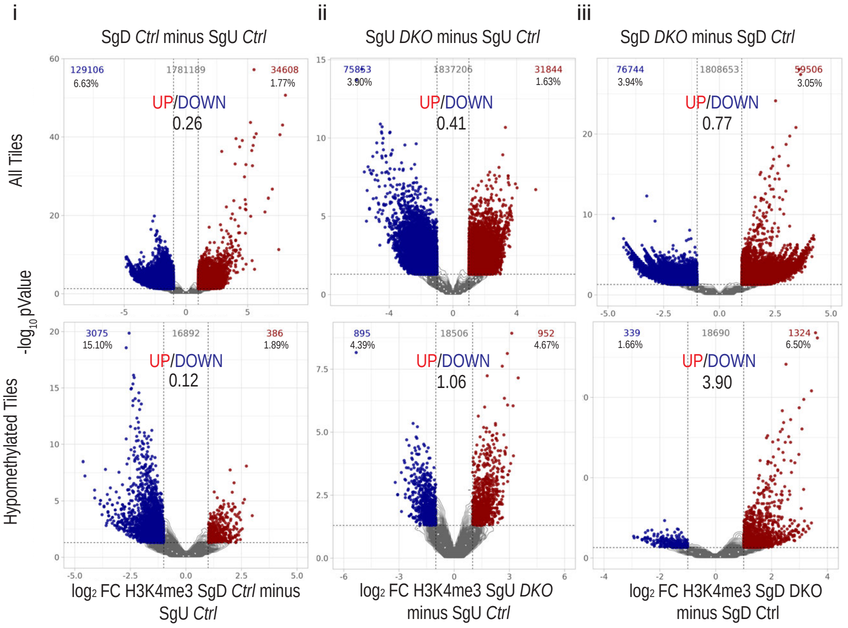
i
ii
iii
SgD Ctrl minus SgU Ctrl
SgU DKO minus SgU Ctrl
SgD DKO minus SgD Ctrl
All Tiles
Hypomethylated Tiles
-log10 pValue
129106
6.63%
1781189
34608
1.77%
UP/DOWN
0.26
75853
3.90%
1837206
31844
1.63%
UP/DOWN
0.41
76744
3.94%
1808653
59506
3.05%
UP/DOWN
0.77
3075
15.10%
16892
386
1.89%
UP/DOWN
0.12
895
4.39%
18506
952
4.67%
UP/DOWN
1.06
339
1.66%
18690
1324
6.50%
UP/DOWN
3.90
log2 FC H3K4me3 SgD Ctrl minus SgU Ctrl
log2 FC H3K4me3 SgU DKO minus SgU Ctrl
log2 FC H3K4me3 SgD DKO minus SgD Ctrl

C

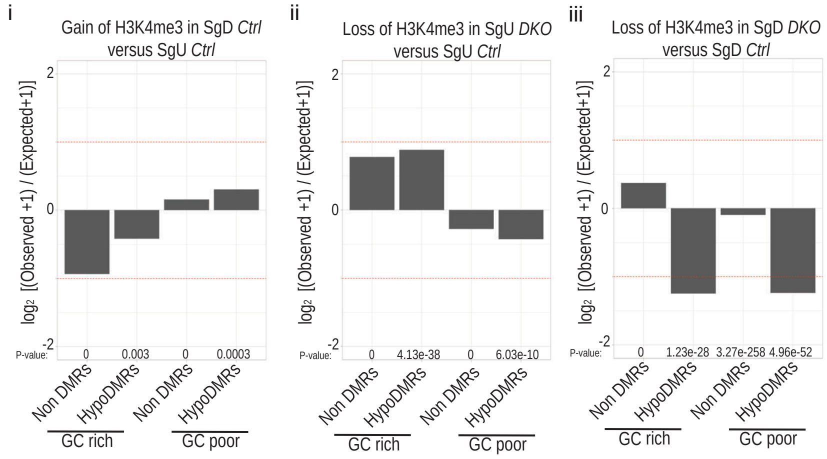
i
ii
iii
Gain of H3K4me3 in SgD Ctrl versus SgU Ctrl
Loss of H3K4me3 in SgU DKO versus SgU Ctrl
Loss of H3K4me3 in SgD DKO versus SgD Ctrl
log2 [(Observed +1) / (Expected+1)]
P-value:
0
0.003
0
0.0003
0
4.13e-38
0
6.03e-10
0
1.23e-28
3.27e-258
4.96e-52
Non DMRs
HypoDMRs
Non DMRs
HypoDMRs
GC rich
GC poor

Supplementary Figure 9. Differential H3K4me3 analysis in *DKO* spermatogonia.

A) Scatterplots [left side] show the H3K4me3 log_2RPKM values at 100.000 randomly selected genomic tiles detected by ULI-NChIP-seq between pairs of biological replicated samples of undifferentiated spermatogonia (SgU), differentiated spermatogonia (SgD) from *Ctrl* and *DKO* animals. The diagonal line plots show the distribution of the H3K4me3 log_2RPKM values in each replicate. The Pearson correlation coefficient [right side] of H3K4me3 log_2RPKM values at 100.000 randomly selected genomic tiles detected by ULI-NChIP-seq between the biological replicates of undifferentiated spermatogonia (SgU), differentiated spermatogonia (SgD) from *Ctrl* and *DKO* samples.

B) Volcano plots showing differential enrichment for H3K4me3 in (i) *Ctrl* SgD (n=2) versus *Ctrl* SgU (n=2), (ii) *DKO* SgU (n=2) versus *Ctrl* SgU (n=2) and (iii) *DKO* SgD (n=2) versus *Ctrl* SgD (n=2) (right) contrasts for all genomic tiles (upper) and hypoDMR tiles (lower). Tiles with statistically significant gain and loss of the H3K4me3 signals are highlighted in red (upregulated) and blue (downregulated) and their numbers and percentage of differentially are shown on the upper right and left corners respectively. The ratio between upregulated versus downregulated tiles in each plot is shown in the middle.

C) Bar plots showing log_2 enrichments of GC-rich or GC-poor nonDMR and hypoDMR tiles among tiles with (i) significant H3K4me3 gain in *Ctrl* SgD versus *Ctrl* SgU, (ii) significant H3K4me3 loss in *DKO* SgU versus *Ctrl* SgU and (iii) significant H3K4me3 loss in *DKO* SgD versus *Ctrl* SgD. P-values were calculated by Fisher's exact test.

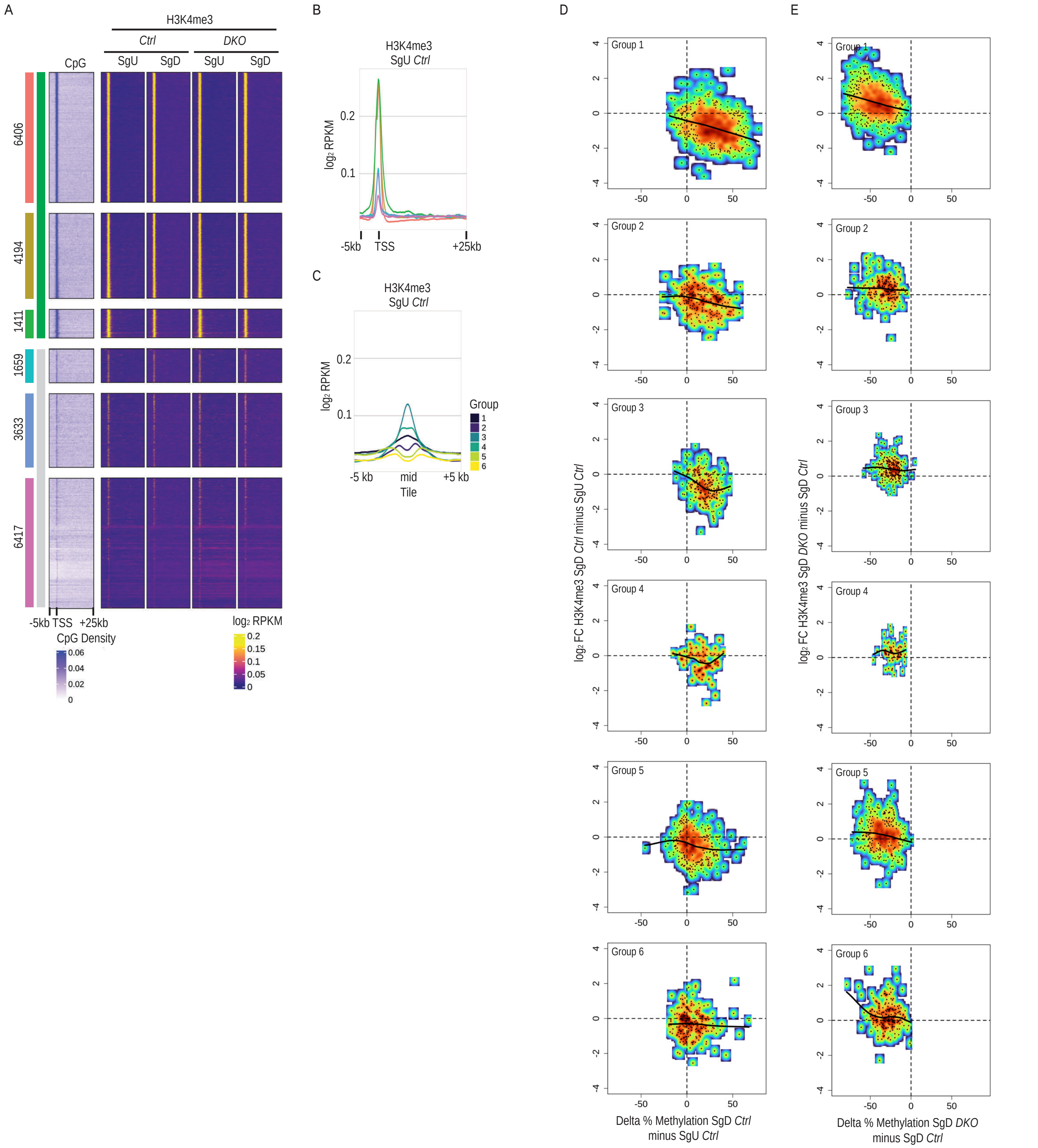
A
H3K4me3
Ctrl
DKO
CpG
SgU
SgD
SgU
SgD
6406
4194
1411
1659
3633
6417
-5kb TSS +25kb
CpG Density
0.06
0.04
0.02
0
log2 RPKM
0.2
0.15
0.1
0.05
0
B
H3K4me3
SgU Ctrl
log2 RPKM
0.2
0.1
-5kb
TSS
+25kb
C
H3K4me3
SgU Ctrl
log2 RPKM
0.2
0.1
Group
1
2
3
4
5
6
-5 kb
mid
+5 kb
Tile
D
Group 1
Group 2
Group 3
Group 4
Group 5
Group 6
log2 FC H3K4me3 SgD Ctrl minus SgU Ctrl
Delta % Methylation SgD Ctrl minus SgU Ctrl
E
log2 FC H3K4me3 SgD DKO minus SgD Ctrl
Delta % Methylation SgD DKO minus SgD Ctrl

Supplementary Figure 10. H3K4me3 levels of hypoDMRs in *DKO* spermatogonia.

A) Heatmaps showing H3K4me3 occupancy at gene transcriptional start sites (TSS) (-5/+25kb) in *Ctrl* and *DKO* SgU and SgD (n=2 for each sample). The CpG density at gene TSS (-5/+25kb) is indicated. Genes have been grouped according to the presence of a CpG island at their TSS +/- 1kb and ordered by decreasing expression in *Ctrl* SgU.

B) Line plots showing average H3K4me3 occupancy at gene TSS (-5/+25kb) in *Ctrl* SgU (n=2) for each expression group.

C) Line plots showing average H3K4me3 occupancy at Groups 1-6 hypoDMRs (±5kb) in *Ctrl* SgU (n=2).

D) Scatterplots showing DNAme difference between *Ctrl* SgD minus *Ctrl* SgU (x-axis) versus $\log_2$ fold change of H3K4me3 signal between *Ctrl* SgD minus *Ctrl* SgU (y-axis) for hypoDMRs in Groups 1-6. Black lines represent LOESS weighted linearly regressed data.

E) Scatterplots showing DNAme difference between *DKO* SgD minus *Ctrl* SgD (x-axis) versus $\log_2$ fold change of H3K4me3 signal between *DKO* SgD minus *Ctrl* SgD (y-axis) for hypoDMRs in Groups 1-6. Black lines represent LOESS weighted linearly regressed data.

Fanourgakis et al Supplementary Figure 11

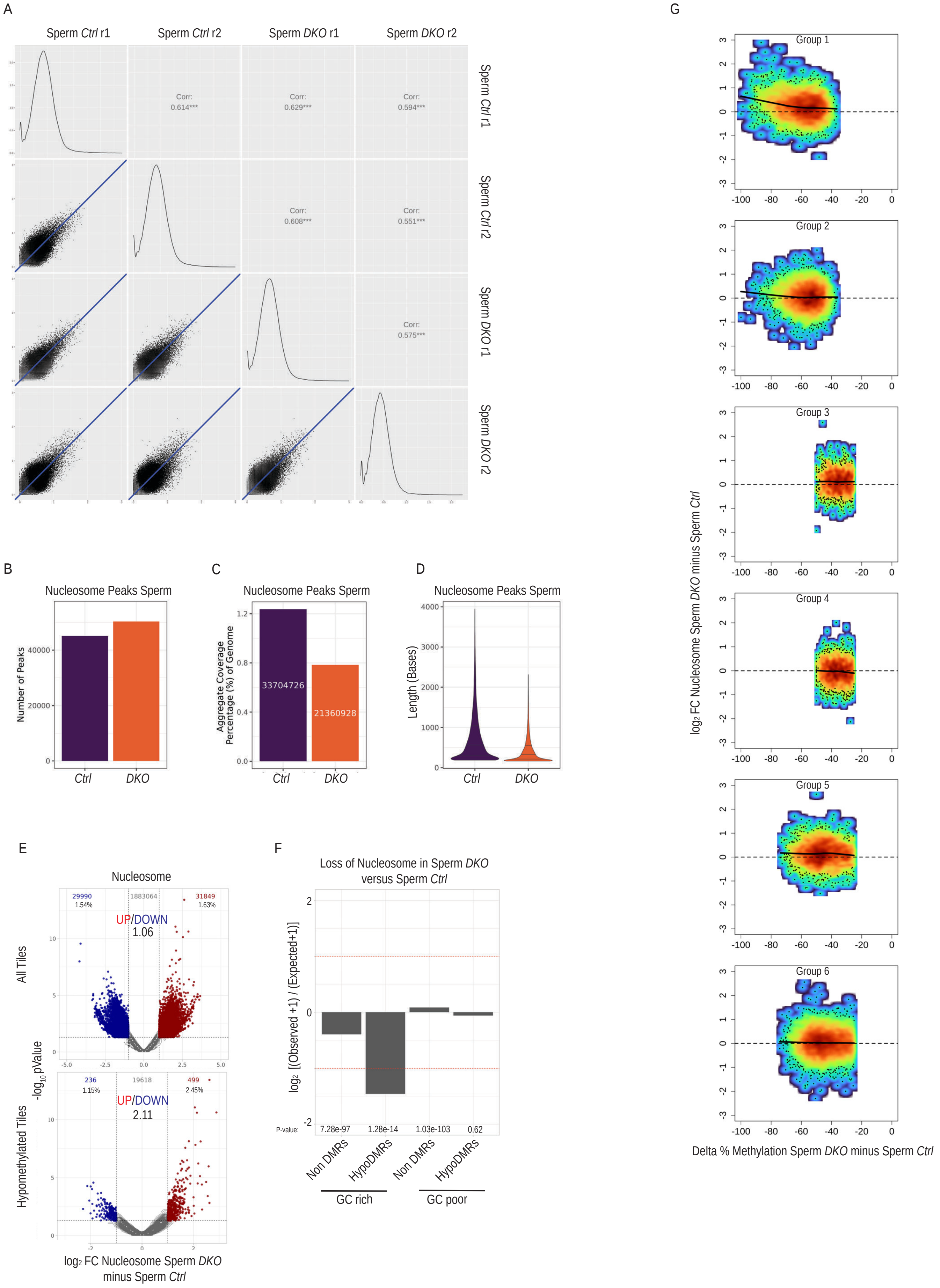

Supplementary Figure 11. Differential nucleosome analysis in *DKO* sperm.

A) Scatterplots [left side] show the nucleosome log_2RPKM values at 100.000 randomly selected genomic tiles detected by ULI-NChIP-seq between pairs of biological replicated samples of sperm from *Ctrl* and *DKO* animals. The diagonal line plots show the distribution of the nucleosome log_2RPKM values in each replicate. The Pearson correlation coefficient [right side] of nucleosome log_2RPKM values at 100.000 randomly selected genomic tiles detected by ULI-NChIP-seq between the biological replicates of sperm from *Ctrl* and *DKO* samples.

B) Bar plot showing the number of anti-Nucleosome ChIP peaks detected by MACS3 in in *Ctrl* (n=2) and *DKO* sperm (n=2) samples.

C) Bar plot showing the percentage of genome covered by anti-Nucleosome ChIP peaks detected by MACS3 in in *Ctrl* (n=2) and *DKO* sperm (n=2) samples. The absolute number of covered bases are shown for each bar.

D) Violin plot showing the length distribution of anti-Nucleosome ChIP peaks detected by MACS3 in in *Ctrl* (n=2) and *DKO* sperm (n=2) samples. Center lines in violin plots represent median values while upper and lower lines indicate the interquartile range (IQR; from the 25th to 75th percentile).

E) Volcano plots showing differential enrichment for nucleosome in *DKO* (n=2) versus *Ctrl* (n=2) sperm contrast for all genomic tiles (upper) and hypoDMR tiles (lower). Tiles with statistically significant gain and loss of the nucleosomal signals are highlighted in red (upregulated) and blue (downregulated) and their numbers and percentage of differentially are shown on the upper right and left corners respectively. The ratio between upregulated versus downregulated tiles in each plot is shown in the middle.

F) Bar plots showing log_2 enrichments of GC-rich or GC-poor nonDMR and hypoDMR tiles among tiles with significant nucleosome loss in *DKO* sperm versus *Ctrl*. P-values were calculated by Fisher's exact test.

G) Scatterplots showing DNAme difference between *DKO* minus *Ctrl* sperm (x-axis) versus log_2 fold change of nucleosome signal between *DKO* minus *Ctrl* sperm (y-axis) for hypoDMRs in Groups 1-6. Black lines represent LOESS weighted linearly regressed data.

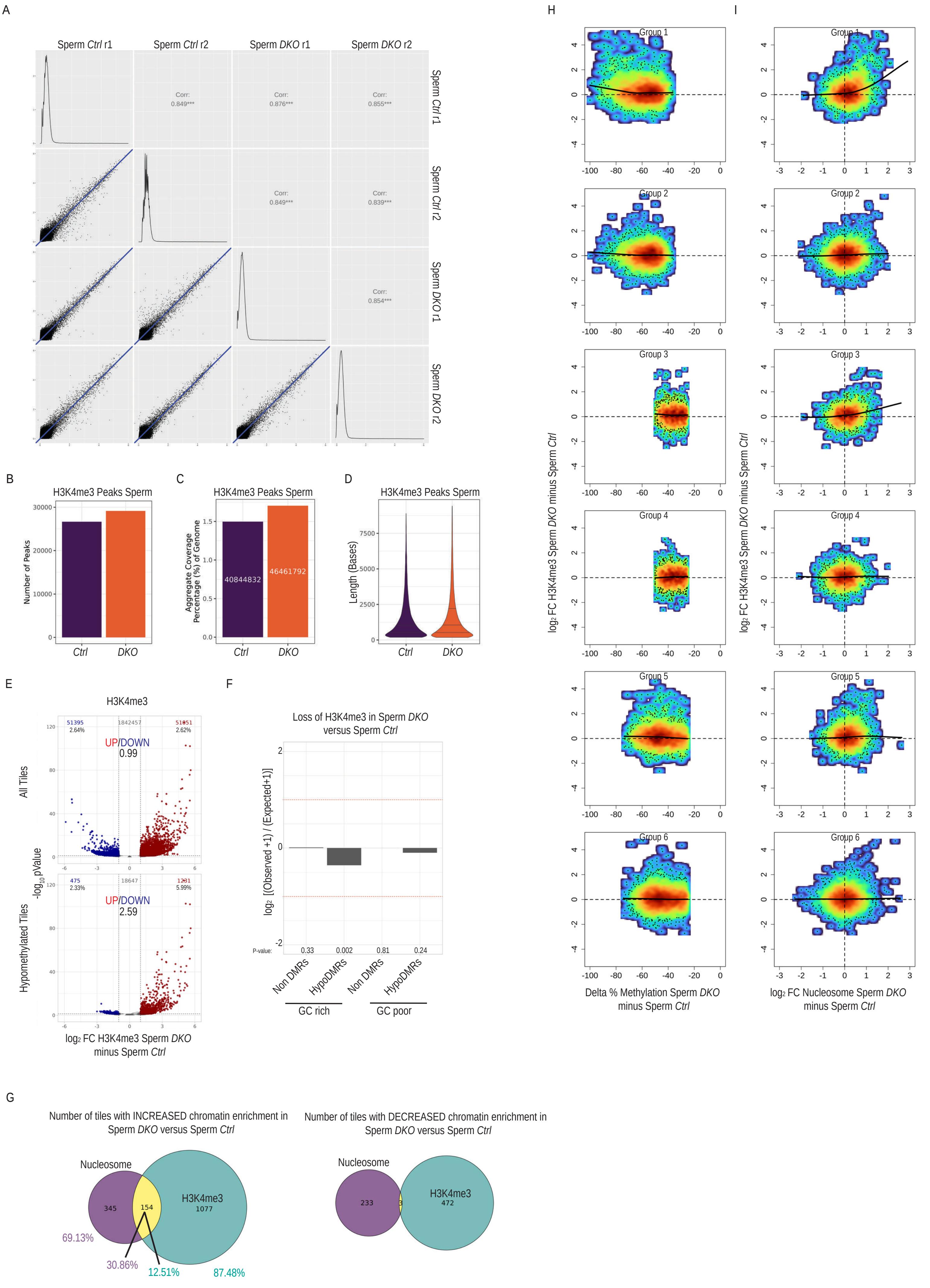
A
Sperm Ctrl r1
Sperm Ctrl r2
Sperm DKO r1
Sperm DKO r2
Corr: 0.849***
Corr: 0.876***
Corr: 0.855***
Corr: 0.849***
Corr: 0.839***
Corr: 0.854***
B
H3K4me3 Peaks Sperm
Number of Peaks
Ctrl
DKO
C
H3K4me3 Peaks Sperm
Aggregate Coverage Percentage (%) of Genome
40844832
46461792
Ctrl
DKO
D
H3K4me3 Peaks Sperm
Length (Bases)
Ctrl
DKO
E
H3K4me3
All Tiles
Hypomethylated Tiles
-log10 pValue
51395 2.64%
1842457
51851 2.62%
UP/DOWN 0.99
475 2.33%
18647
1231 5.99%
UP/DOWN 2.59
log2 FC H3K4me3 Sperm DKO minus Sperm Ctrl
F
Loss of H3K4me3 in Sperm DKO versus Sperm Ctrl
log2 [(Observed +1) / (Expected+1)]
P-value:
0.33
0.002
0.81
0.24
Non DMRs
HypoDMRs
Non DMRs
HypoDMRs
GC rich
GC poor
G
Number of tiles with INCREASED chromatin enrichment in Sperm DKO versus Sperm Ctrl
Nucleosome
H3K4me3
345
154
1077
69.13%
30.86%
12.51%
87.48%
Number of tiles with DECREASED chromatin enrichment in Sperm DKO versus Sperm Ctrl
Nucleosome
H3K4me3
233
472
H
I
Group 1
Group 2
Group 3
Group 4
Group 5
Group 6
log2 FC H3K4me3 Sperm DKO minus Sperm Ctrl
Delta % Methylation Sperm DKO minus Sperm Ctrl
log2 FC Nucleosome Sperm DKO minus Sperm Ctrl

Supplementary Figure 12. Differential H3K4me3 analysis in *DKO* sperm.

A) Scatterplots [left side] show the H3K4me3 log_2RPKM values at 100.000 randomly selected genomic tiles detected by ULI-NChIP-seq between pairs of biological replicated samples of sperm from *Ctrl* and *DKO* animals. The diagonal line plots show the distribution of the H3K4me3 log_2RPKM values in each replicate. The Pearson correlation coefficient [right side] of H3K4me3 log_2RPKM values at 100.000 randomly selected genomic tiles detected by ULI-NChIP-seq between the biological replicates of sperm from *Ctrl* and *DKO* samples.

B) Bar plot showing the number of anti-H3K4me3 ChIP peaks detected by MACS3 in in *Ctrl* (n=2) and *DKO* sperm (n=2) samples.

C) Bar plot showing the percentage of genome covered by anti-H3K4me3 ChIP peaks detected by MACS3 in in *Ctrl* (n=2) and *DKO* sperm (n=2) samples. The absolute number of covered bases are shown for each bar.

D) Violin plot showing the length distribution of anti-H3K4me3 ChIP peaks detected by MACS3 in in *Ctrl* (n=2) and *DKO* sperm (n=2) samples. Center lines in violin plots represent median values while upper and lower lines indicate the interquartile range (IQR; from the 25th to 75th percentile).

E) Volcano plots showing differential enrichment for H3K4me3 in *DKO* (n=2) versus *Ctrl* (n=2) sperm contrast for all genomic tiles (upper) and hypoDMR tiles (lower). Tiles with statistically significant gain and loss of the nucleosomal signals are highlighted in red (upregulated) and blue (downregulated) and their numbers and percentage of differentially are shown on the upper right and left corners respectively. The ratio between upregulated versus downregulated tiles in each plot is shown in the middle.

F) Bar plots showing log_2 enrichments of GC-rich or GC-poor nonDMR and hypoDMR tiles among tiles with significant H3K4me3 loss in *DKO* sperm versus *Ctrl*. P-values were calculated by Fisher's exact test.

G) Euler diagrams representing the number of hypoDMRs that are differentially gain enrichment in *DKO* versus *Ctrl* sperm either for nucleosome or H3K4me3 or both (left) and the number of hypoDMRs that are differentially lose enrichment in *DKO* versus *Ctrl* sperm either for nucleosome or H3K4me3 or both (right).

H) Scatterplots showing DNAme difference between *DKO* minus *Ctrl* sperm (x-axis) versus log_2 fold change of H3K4me3 signal between *DKO* minus *Ctrl* sperm (y-axis) for hypoDMRs in Groups 1-6. Black lines represent LOESS weighted linearly regressed data.

I) Scatterplots showing log_2 fold change of nucleosome signal between *DKO* minus *Ctrl* sperm (x-axis) versus log_2 fold change of H3K4me3 signal between *DKO* minus *Ctrl* sperm (y-axis) for hypoDMRs in Groups 1-6. Black lines represent LOESS weighted linearly regressed data.

Fanourgakis et al Supplementary Figure 13

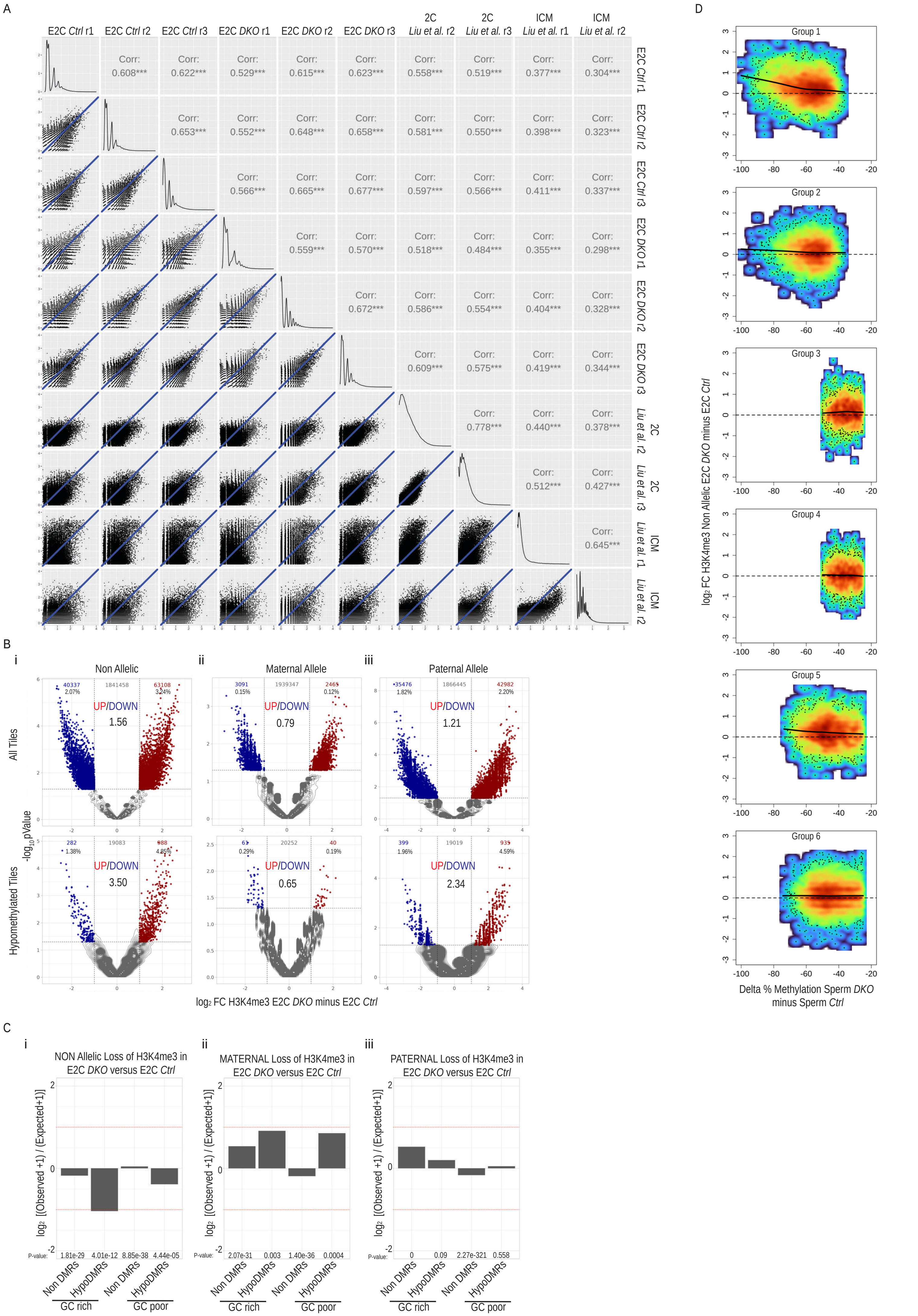

Supplementary Figure 13. Differential H3K4me3 analysis in *DKO* sperm derived early 2 cell stage (E2C) embryos.

A) Scatterplots [left side] show the H3K4me3 log_2RPKM values at 100.000 randomly selected genomic tiles detected by ATATA-seq between pairs of biological replicated samples of early 2-cell stage (E2C) embryos generated by *Ctrl* or *DKO* sperm and detected by CHIP-seq in wild type 2 cell stage embryos and blastocyst stage ICM samples (from Liu et al., 2016) as a reference. The diagonal line plots show the distribution of the H3K4me3 log_2RPKM values in each replicate. The Pearson correlation coefficient [right side] of H3K4me3 log_2RPKM values at 100.000 randomly selected genomic tiles detected by ATATA-seq between the biological replicated samples of early 2-cell stage (E2C) embryos generated by *Ctrl* or *DKO* sperm and detected by CHIP-seq in wild type 2 cell stage embryos and blastocyst stage ICM samples (from Liu et al., 2016) as a reference.

B) Volcano plots showing differential enrichment for H3K4me3 in *DKO* (n=3) versus *Ctrl* (n=3) sperm derived E2C embryos contrast (i) without allelic assignment, (ii) at the maternal allele or (iii) at the paternal allele for all genomic tiles (upper) and hypoDMR tiles (lower). Tiles with statistically significant gain and loss of the H3K4me3 signals are highlighted in red (upregulated) and blue (downregulated) and their numbers and percentage of differentially are shown on the upper right and left corners respectively. The ratio between upregulated versus downregulated tiles in each plot is shown in the middle.

C) Bar plots showing log2 enrichments of GC-rich or GC-poor nonDMR and hypoDMR tiles among tiles with significant H3K4me3 loss at (i) all alleles, (ii) maternal alleles and (iii) paternal alleles in early 2-cell (E2C) embryos generated by *Ctrl* or *DKO* sperm. P-values were calculated by Fisher's exact test.

D) Scatterplots showing DNAme difference between *DKO* minus *Ctrl* sperm (x-axis) versus log2 fold change of H3K4me3 signal between *DKO* minus *Ctrl* sperm derived early 2-cell (E2C) embryos (y-axis) for hypoDMRs in Groups 1-6. Black lines represent LOESS weighted linearly regressed data.

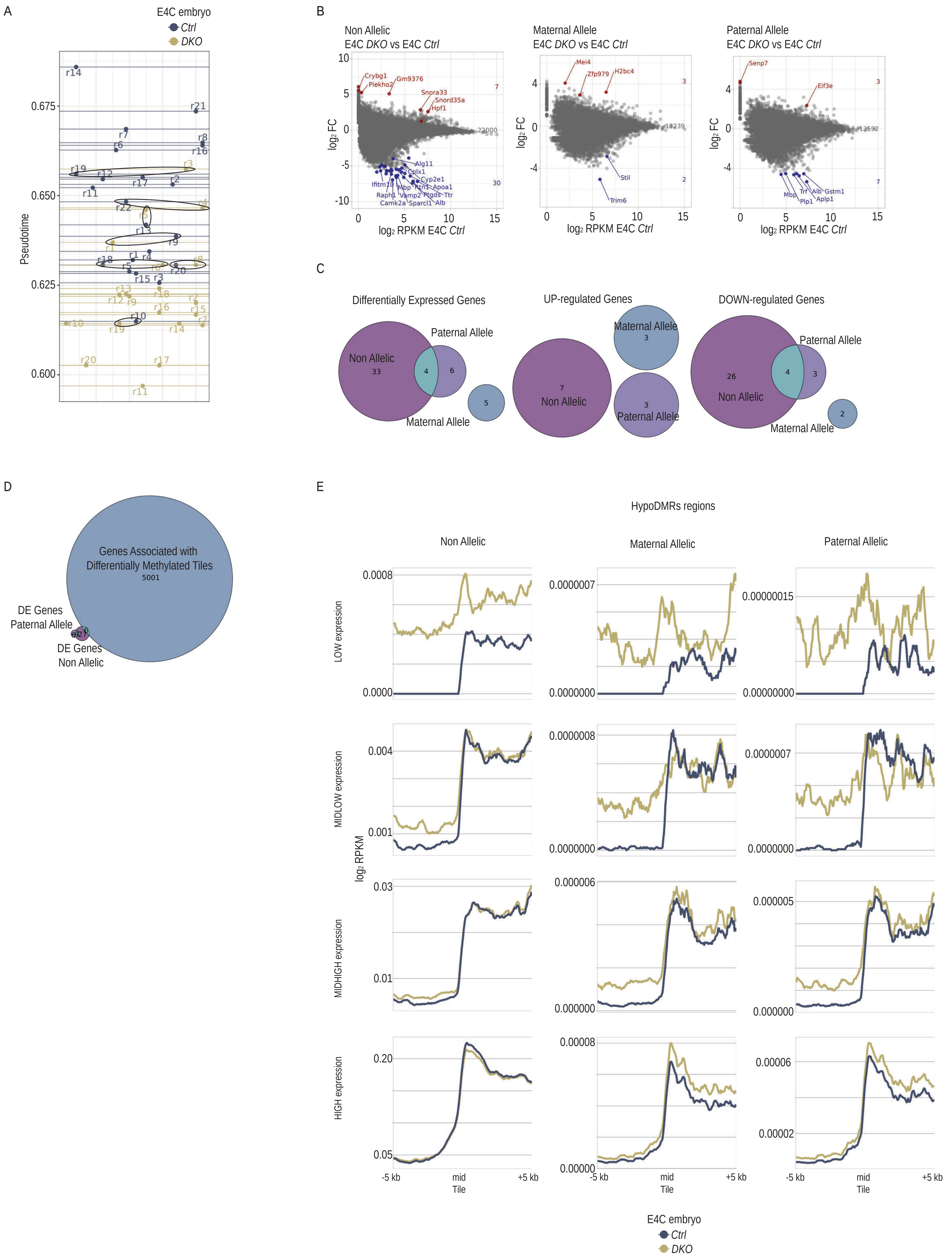
A
E4C embryo
Ctrl
DKO
Pseudotime
0.675
0.650
0.625
0.600
B
Non Allelic
E4C DKO vs E4C Ctrl
Maternal Allele
E4C DKO vs E4C Ctrl
Paternal Allele
E4C DKO vs E4C Ctrl
log2 FC
log2 RPKM E4C Ctrl
Crybg1
Plekho2
Gm9376
Snora33
Snord35a
Hpf1
Alg11
Colx1
Cyp2e1
Ifitm10
Mbp
Ptn1
Apoa1
Raph1
Vamp2
Ptgds
Ttr
Camk2a
Sparcl1
Alb
22000
30
7
Mei4
Zfp979
H2bc4
Stil
Trim6
13239
Senp7
Eif3e
Trf
Alb
Gstm1
Plp1
Aplp1
13592
C
Differentially Expressed Genes
Paternal Allele
Non Allelic
33
4
6
5
Maternal Allele
UP-regulated Genes
Maternal Allele
3
7
Non Allelic
3
Paternal Allele
DOWN-regulated Genes
Paternal Allele
26
4
3
Non Allelic
2
Maternal Allele
D
Genes Associated with
Differentially Methylated Tiles
5001
DE Genes
Paternal Allele
DE Genes
Non Allelic
E
HypoDMRs regions
Non Allelic
Maternal Allelic
Paternal Allelic
LOW expression
MIDLOW expression
MIDHIGH expression
HIGH expression
log2 RPKM
-5 kb
mid
+5 kb
Tile
E4C embryo
Ctrl
DKO

Supplementary Figure 14. Transcriptional effects of *Dnmt3a*/*Dnmt3b* *DKO* and *Ctrl* sperm derived embryos.

A) Dotplot shows the assigned developmental pseudotime of each embryo (n= 21 for *Ctrl* and n= 20 for *DKO* sperm derived embryos). Circles denote paired samples that were used for downstream differential gene expression analysis (n= 7 for *Ctrl* and n= 7 for *DKO* sperm derived embryos).

B) Scatter plots showing gene expression log_2 fold changes among *DKO* (n=7) versus *Ctrl* (n=7) sperm derived 4-cell stage embryos samples were plotted against average gene expression levels in *Ctrl* sperm derived 4-cell stage embryos samples. From left to right: using all reads regardless parental origin, reads from maternal allele, reads from paternal allele. The numbers of significantly Upregulated (red) or Downregulated (blue) genes are displayed on the left and genes names of the most highly significant differentially expressed genes are shown.

C) Euler diagrams representing the number of differentially expressed genes from (B) for non-allelic, maternal, or paternal allele specific analysis (left), the number of upregulated genes from (B) for non-allelic, maternal or paternal allele specific analysis (middle) and the number of downregulated genes from (B) for non-allelic, maternal or paternal allele specific analysis (right).

D) Euler diagram representing the number of differentially expressed genes from (B) for non-allelic, maternal, or paternal allele specific analysis and the number of hypoDMR regions associated genes.

E) Line plots showing average RNA occupancy at the center of hypoDMR regions (±5 kb) in *DKO* versus *Ctrl* sperm derived 4 cell stage embryos samples. The hypoDMR regions are oriented to display high occupancy on the positive direction and stratified into 4 quantile expression groups (low, midlow, midhigh and high) based in the *Ctrl* sperm derived 4 cell stage embryos. From left to right: using all reads regardless parental origin, reads from maternal allele, reads from paternal allele.

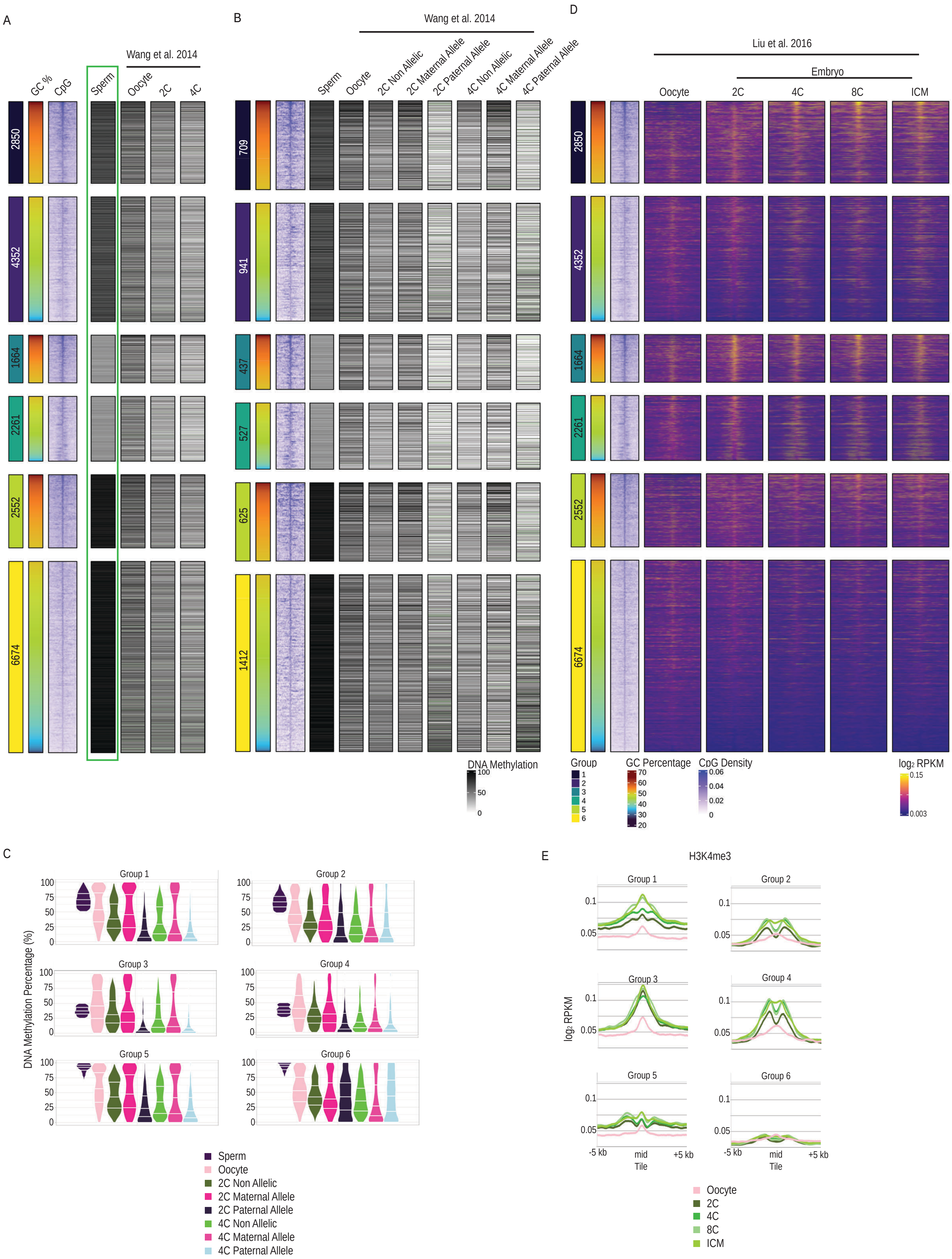
A
GC %
CpG
Sperm
Oocyte
2C
4C
Wang et al. 2014
2850
4352
1664
2261
2552
6674
B
Wang et al. 2014
Sperm
Oocyte
2C Non Allelic
2C Maternal Allele
2C Paternal Allele
4C Non Allelic
4C Maternal Allele
4C Paternal Allele
709
941
437
527
625
1412
DNA Methylation
100
50
0
D
Liu et al. 2016
Embryo
Oocyte
2C
4C
8C
ICM
2850
4352
1664
2261
2552
6674
Group
1
2
3
4
5
6
GC Percentage
70
60
50
40
30
20
CpG Density
0.06
0.04
0.02
0
log2 RPKM
0.15
0.003
C
DNA Methylation Percentage (%)
Group 1
Group 2
Group 3
Group 4
Group 5
Group 6
100
75
50
25
0
Sperm
Oocyte
2C Non Allelic
2C Maternal Allele
2C Paternal Allele
4C Non Allelic
4C Maternal Allele
4C Paternal Allele
E
H3K4me3
log2 RPKM
Group 1
Group 2
Group 3
Group 4
Group 5
Group 6
0.1
0.05
-5 kb
mid
+5 kb
Tile
Oocyte
2C
4C
8C
ICM

Supplementary Figure 15. DNAme and H3K4me3 dynamics of hypoDMRs in *Ctrl* early embryogenesis.

A) Heatmap showing the DNAme percentage of hypoDMRs in *Ctrl* sperm (this study), wild type oocytes, 2-cell stage and 4 cell stage embryos (from Wang et al., 2014). The density of CpGs at hypoDMRs (±5 kb from their center) is indicated. Within each group, hypoDMRs are ordered according to decreasing GC percentages. The panel in the green box is presented also in Figure 1E.

B) Heatmap showing the DNAme percentage as in (A), but for the subset of hypoDMRs that contain enough informative SNPs to allow parental allelic assignment. From left to right: *Ctrl* sperm (this study), wild type oocytes, 2-cell stage without genome assignment, 2-cell stage maternal allele, 2-cell stage paternal allele, 4-cell stage without genome assignment, 4-cell stage maternal allele and 4-cell stage paternal allele (from Wang et al., 2014). The density of CpGs at hypoDMRs (±5 kb from their center) is indicated. Within each group, hypoDMRs are ordered according to decreasing GC percentages.

C) Violin plots showing the percentage of DNAme of hypoDMRs in *Ctrl* sperm (this study), wild type oocytes, 2-cell stage without genome assignment, 2-cell stage maternal allele, 2-cell stage paternal allele, 4-cell stage without genome assignment, 4-cell stage maternal allele and 4-cell stage paternal allele (from Wang et al., 2014) for each group. The center lines in the violins represent median values. The upper and lower lines indicate the interquartile range (IQR; from the 25th to 75th percentile).

D) Heatmaps showing H3K4me3 occupancy at center of Group 1 to 6 hypoDMRs (±5kb) in wild type oocytes, 2-cell stage embryos, 4-cell stage embryos, 8-cell stage embryos and ICM blastocyst stage embryos (from Liu et al., 2016). The density of CpGs at hypoDMRs (±5 kb from their center) is indicated. Within each group, hypoDMRs are ordered according to decreasing GC percentages.

E) Line plots showing average H3K4me3 occupancy at Groups 1-6 hypoDMRs (±5kb) in wild type oocytes, 2-cell stage embryos, 4-cell stage embryos, 8-cell stage embryos and ICM blastocyst stage embryos (from Liu et al., 2016).
