# Supplementary material for: DNA methylation modulates nucleosome retention in sperm and H3K4 methylation deposition in early mouse embryos
Source: Nat Commun. 2025 Jan 7;16:465. doi: 10.1038/s41467-024-55441-1 (PMC11706963; doi:10.1038/s41467-024-55441-1)
Supplement: Supplementary file 3 — Reporting Summary [file 41467_2024_55441_MOESM3_ESM.pdf]

nature portfolio

Corresponding author(s): Antoine HFM Peters

Last updated by author(s): Nov 4, 2024

Reporting Summary

Nature Portfolio wishes to improve the reproducibility of the work that we publish. This form provides structure for consistency and transparency in reporting. For further information on Nature Portfolio policies, see our Editorial Policies and the Editorial Policy Checklist.

Statistics

For all statistical analyses, confirm that the following items are present in the figure legend, table legend, main text, or Methods section.

n/a	Confirmed	
☐	☒	The exact sample size (*n*) for each experimental group/condition, given as a discrete number and unit of measurement
☐	☒	A statement on whether measurements were taken from distinct samples or whether the same sample was measured repeatedly
☐	☒	The statistical test(s) used AND whether they are one- or two-sided<br>*Only common tests should be described solely by name; describe more complex techniques in the Methods section.*
☐	☒	A description of all covariates tested
☐	☒	A description of any assumptions or corrections, such as tests of normality and adjustment for multiple comparisons
☐	☒	A full description of the statistical parameters including central tendency (e.g. means) or other basic estimates (e.g. regression coefficient) AND variation (e.g. standard deviation) or associated estimates of uncertainty (e.g. confidence intervals)
☐	☒	For null hypothesis testing, the test statistic (e.g. *F*, *t*, *r*) with confidence intervals, effect sizes, degrees of freedom and *P* value noted<br>*Give P values as exact values whenever suitable.*
☒	☐	For Bayesian analysis, information on the choice of priors and Markov chain Monte Carlo settings
☒	☐	For hierarchical and complex designs, identification of the appropriate level for tests and full reporting of outcomes
☐	☒	Estimates of effect sizes (e.g. Cohen's *d*, Pearson's *r*), indicating how they were calculated

Our web collection on statistics for biologists contains articles on many of the points above.

Software and code

Policy information about availability of computer code

Data collection	Genomic data were collected with HiSeq 2500 (Illumina) and NextSeq 500 (Illumina) platforms.<br>Imaging data were collected with CSU W1 Dual T2 (Yokogawa) and Axioscan Z1 (Zeiss) microscopes.<br>Flow cytometry data were collected with BD FACSAria III (Becton Dickinson) cell sorter.
Data analysis	TrimGalore (version 0.6.2), STAR (version 2.5.0a), Bismark (version 0.23.3), SAMtools (version 1.10), nudup.py (version 2.2), macs3 (version 3.0.0bl), Rstudio (R version 4.2.1), limma (version 3.52.2), GenomicFeatures (version 1.56.0), QuasR (version 1.36.0), SCORPIUS (vl.0.8), PCAtools (version 2.8.0), stats (version 4.2.1) , topGO (version 2.48.0), monalisa (version 1.10.1), edgeR (version 3.42.4), lmageJ (Fiji version l.53f51), FACS BD Diva (version 9), FlowJo (version 10)

For manuscripts utilizing custom algorithms or software that are central to the research but not yet described in published literature, software must be made available to editors and reviewers. We strongly encourage code deposition in a community repository (e.g. GitHub). See the Nature Portfolio guidelines for submitting code & software for further information.

Data

Policy information about availability of data

All manuscripts must include a data availability statement. This statement should provide the following information, where applicable:

- Accession codes, unique identifiers, or web links for publicly available datasets
- A description of any restrictions on data availability
- For clinical datasets or third party data, please ensure that the statement adheres to our policy

The genomic datasets produced in this study are available in the following database: Gene Expression Omnibus GSE229246 (https://www.ncbi.nlm.nih.gov/geo/query/acc.cgi?acc=GSE229246). Publicly available datasets used in this study can be found at Gene Expression Omnibus database with the following accession IDs: GSE148150, GSE56697 and GSE73952. Imaging and flow cytometry data are available from the corresponding author on reasonable request.

Research involving human participants, their data, or biological material

Policy information about studies with human participants or human data. See also policy information about sex, gender (identity/presentation), and sexual orientation and race, ethnicity and racism.

Reporting on sex and gender	*Use the terms sex (biological attribute) and gender (shaped by social and cultural circumstances) carefully in order to avoid confusing both terms. Indicate if findings apply to only one sex or gender; describe whether sex and gender were considered in study design; whether sex and/or gender was determined based on self-reporting or assigned and methods used.*<br>*Provide in the source data disaggregated sex and gender data, where this information has been collected, and if consent has been obtained for sharing of individual-level data; provide overall numbers in this Reporting Summary. Please state if this information has not been collected.*<br>*Report sex- and gender-based analyses where performed, justify reasons for lack of sex- and gender-based analysis.*
Reporting on race, ethnicity, or other socially relevant groupings	*Please specify the socially constructed or socially relevant categorization variable(s) used in your manuscript and explain why they were used. Please note that such variables should not be used as proxies for other socially constructed/relevant variables (for example, race or ethnicity should not be used as a proxy for socioeconomic status).*<br>*Provide clear definitions of the relevant terms used, how they were provided (by the participants/respondents, the researchers, or third parties), and the method(s) used to classify people into the different categories (e.g. self-report, census or administrative data, social media data, etc.)*<br>*Please provide details about how you controlled for confounding variables in your analyses.*
Population characteristics	*Describe the covariate-relevant population characteristics of the human research participants (e.g. age, genotypic information, past and current diagnosis and treatment categories). If you filled out the behavioural & social sciences study design questions and have nothing to add here, write "See above."*
Recruitment	*Describe how participants were recruited. Outline any potential self-selection bias or other biases that may be present and how these are likely to impact results.*
Ethics oversight	*Identify the organization(s) that approved the study protocol.*

Note that full information on the approval of the study protocol must also be provided in the manuscript.

Field-specific reporting

Please select the one below that is the best fit for your research. If you are not sure, read the appropriate sections before making your selection.

☒ Life sciences ☐ Behavioural & social sciences ☐ Ecological, evolutionary & environmental sciences

For a reference copy of the document with all sections, see nature.com/documents/nr-reporting-summary-flat.pdf

Life sciences study design

All studies must disclose on these points even when the disclosure is negative.

Sample size	For genomic experiments at least 2 independent biological samples were acquired from individual animals. For EMseq, ChIPseq and RRBS, 2 biological replicates for each biological condition were performed. For SMARTseq2, 42 single embryos were generated by IVF using sperm from 2 males and 20 females and analyzed individually. For ATATAseq we perfromed 3 replicates for each biological condition: 280 embryos were generated by IVF using sperm from 4 males and 40 females and pooled in 6 groups of 50-50-45-45-45-45 each. For bulk RNAseq 3 replicates for each biological condition were performed. For histolopathology experiments at least 2 independent biological samples were acquired from individual animals. The sample sizes were determined based on our previous experimental experience in which such numbers could give cost efficient and reliable result for downstream analysis.
Data exclusions	No data were excluded.
Replication	*Describe the measures taken to verify the reproducibility of the experimental findings. If all attempts at replication were successful, confirm this OR if there are any findings that were not replicated or cannot be reproduced, note this and describe why.*

Randomization	Randomization was not relevant for this study.
Blinding	Investigators were blinded to group allocation during imaging and manual counting of defective seminiferous tubules of the histopathology experiment. Blinding was not relevant for the other experimental set up.

Reporting for specific materials, systems and methods

We require information from authors about some types of materials, experimental systems and methods used in many studies. Here, indicate whether each material, system or method listed is relevant to your study. If you are not sure if a list item applies to your research, read the appropriate section before selecting a response.

Materials & experimental systems

n/a	Involved in the study
☐	☒ Antibodies
☒	☐ Eukaryotic cell lines
☒	☐ Palaeontology and archaeology
☐	☒ Animals and other organisms
☒	☐ Clinical data
☒	☐ Dual use research of concern
☒	☐ Plants

Methods

n/a	Involved in the study
☐	☒ ChIP-seq
☐	☒ Flow cytometry
☒	☐ MRI-based neuroimaging

Antibodies

Antibodies used

For immunohistochemistry staining :
anti-cKit (R&D systems AF1356) (1:1000)
anti-DNMT3a (Imgenex IMG-268A) (1:1000)
anti-DNMT3b (Imgenex IMG-184A) (1:1000)
species specific secondary Alexafluor-conjugated antibodies (ThermoFischer Scientific) (1:1000)

For FACS:
anti-CD117/c-kit PE (eBioscience 12-1171-83) (1:200)
anti-CD324/E-Cadherin eFluor 660 (eBioscience 50-3249-82) (1:200)
anti-CD49f/Integrin alpha 6 PE-Cyanine7 (eBioscience 25-0459-82) (1:200)

For ChIPseq:
anti-H3K4me3 (Millipore 17-614) (0.5ug/sample)
anti-H3K36me3 (Cell Signaling #4909) (0.75ug/sample)
anti-H3.3 (Cosmo Bio CE-040B) (1ug/sample)
anti-nucleosome provided by J. van der Vlag (1ug/sample) (Kramers, K., Stemmer, C., Monestier, M., van Bruggen, M.C., Rijke-Schilder, T.P., Hylkema, M.N., Smeenk, R.J., Muller, S. and Berden, J.H. (1996) Specificity of monoclonal anti-nucleosome auto-antibodies derived from lupus mice. J Autoimmun, 9, 723-729)
anti-H3.1/2/t provided by J. van der Vlag (1ug/sample) (van der Heijden, G.W., Dieker, J.W., Derijck, A.A., Muller, S., Berden, J.H., Braat, D.D., van der Vlag, J. and de Boer, P. (2005) Asymmetry in histone H3 variants and lysine methylation between paternal and maternal chromatin of the early mouse zygote. Mech Dev, 122, 1008-1022)

ForATATAseq:
anti-H3K4me3 (Millipore 17-614) (1ug/sample)

Validation

anti-cKit (R&D systems AF1356) :
Applications: Western Blot, Simple Western, Flow Cytometry, Immunohistochemistry,CyTOF-ready

anti-DNMT3a (Imgenex IMG-268A) :
Applications: WB, RIP, IP, IHC, IF, ChIP, ELISA

anti-DNMT3b (Imgenex IMG-184A):
Applications: western blot, immunohistochemistry, immunohistochemistry - paraffin section

anti-CD117/c-kit PE (eBioscience 12-1171-83) :
Applications: Immunohistochemistry (Frozen) (IHC (F)), Flow Cytometry (Flow)

anti-CD324/E-Cadherin eFluor 660 (eBioscience 50-3249-82):
Applications: Western Blot (WB), Immunohistochemistry (IHC), Immunocytochemistry (ICC/IF), Flow Cytometry (Flow), Neutralization (Neu)

anti-CD49f/Integrin alpha 6 PE-Cyanine7 (eBioscience 25-0459-82):
Applications: Flow Cytometry (Flow)

anti-H3K4me3 (Millipore 17-614):

Applications: WB, ChIP, ChIP-seq

anti-H3K36me3 (Cell Signaling #4909):
Applications: Western Blotting, Immunohistochemistry (Paraffin), Immunofluorescence (Immunocytochemistry), Flow Cytometry (Fixed/Permeabilized), Chromatin IP, Chromatin IP-seq, CUT&RUN

anti-H3.3 (Cosmo Bio CE-040B):
Applications: Western blotting, Immunocytochemistry, Immunohistochemistry, ChIP, IP

anti-nucleosome Kramers, K., Stemmer, C., Monestier, M., van Bruggen, M.C., Rijke-Schilder, T.P., Hylkema, M.N., Smeenk, R.J., Muller, S. and Berden, J.H. (1996) Specificity of monoclonal anti-nucleosome auto-antibodies derived from lupus mice. J Autoimmun, 9, 723-729

anti-H3.1/2/t van der Heijden, G.W., Dieker, J.W., Derijck, A.A., Muller, S., Berden, J.H., Braat, D.D., van der Vlag, J. and de Boer, P. (2005) Asymmetry in histone H3 variants and lysine methylation between paternal and maternal chromatin of the early mouse zygote. Mech Dev, 122, 1008-1022

Animals and other research organisms

Policy information about studies involving animals; ARRIVE guidelines recommended for reporting animal research, and Sex and Gender in Research

Laboratory animals

Mice of 129SvJae × C57BL/6 hybrid background bearing Dnmt3aflox (Dnmt3atm3.1Enl) and Dnmt3bflox (Dnmt3btm5.1Enl) alleles were provided by Dr. En Li (Okano, M., Bell, D.W., Haber, D.A. and Li, E. (1999) DNA methyltransferases Dnmt3a and Dnmt3b are essential for de novo methylation and mammalian development. Cell, 99, 247-257.).

Mice of C57BL/6 background bearing the iCre expressing transgene under the control of Stra8 promoter (Tg(Stra8-icre)1Reb) were obtained from The Jackson Laboratory (RRID:IMSR_JAX:017490).

JF1/MsJ (Japanese fancy mouse 1) inbred strain was purchased from The Jackson Laboratory (RRID:IMSR_JAX:003720).

C57BL/6JRj mice were purchased from Janvier Labs.

Wild animals

No wild animals used in this study.

Reporting on sex

Male mice were used in this study.

Field-collected samples

No field collected samples used in this study.

Ethics oversight

Swiss Animal Protection Ordinance and FMI institutional ethical committee.

Note that full information on the approval of the study protocol must also be provided in the manuscript.

Plants

Seed stocks

Report on the source of all seed stocks or other plant material used. If applicable, state the seed stock centre and catalogue number. If plant specimens were collected from the field, describe the collection location, date and sampling procedures.

Novel plant genotypes

Describe the methods by which all novel plant genotypes were produced. This includes those generated by transgenic approaches, gene editing, chemical/radiation-based mutagenesis and hybridization. For transgenic lines, describe the transformation method, the number of independent lines analyzed and the generation upon which experiments were performed. For gene-edited lines, describe the editor used, the endogenous sequence targeted for editing, the targeting guide RNA sequence (if applicable) and how the editor was applied.

Authentication

Describe any authentication procedures for each seed stock used or novel genotype generated. Describe any experiments used to assess the effect of a mutation and, where applicable, how potential secondary effects (e.g. second site T-DNA insertions, mosiacism, off-target gene editing) were examined.

ChIP-seq

Data deposition

☒ Confirm that both raw and final processed data have been deposited in a public database such as GEO.

☐ Confirm that you have deposited or provided access to graph files (e.g. BED files) for the called peaks.

Data access links
May remain private before publication.

We submitted genomic data sets to GEO where they are available using the following token: sfgjiygmbnsfpoj. The token provides anonymous, read-only access to GSE229246 and associated accessions (https://www.ncbi.nlm.nih.gov/geo/query/acc.cgi?acc=GSE229246).

Files in database submission

ATATAseq

Files in database submission

GSM7157025 Early 2 cell stage embryos H3K4me3 Dnmt3a f/+ ; Dnmt3b f/+ sperm derived rep 1
GSM7157026 Early 2 cell stage embryos H3K4me3 Dnmt3a f/+ ; Dnmt3b f/+ sperm derived rep 2
GSM7157027 Early 2 cell stage embryos H3K4me3 Dnmt3a f/+ ; Dnmt3b f/+ sperm derived rep 3
GSM7157028 Early 2 cell stage embryos H3K4me3 Dnmt3a f/- ; Dnmt3b f/- ; Stra8-iCre tg sperm derived rep 1
GSM7157029 Early 2 cell stage embryos H3K4me3 Dnmt3a f/- ; Dnmt3b f/- ; Stra8-iCre tg sperm derived rep 2
GSM7157030 Early 2 cell stage embryos H3K4me3 Dnmt3a f/- ; Dnmt3b f/- ; Stra8-iCre tg sperm derived rep 3
GSM7157031 Early 2 cell stage embryos IgG Dnmt3a f/+ ; Dnmt3b f/+ sperm derived rep 1
GSM7157032 Early 2 cell stage embryos IgG Dnmt3a f/- ; Dnmt3b f/- ; Stra8-iCre tg sperm derived rep 1

EMseq
GSM7157111 Sperm Dnmt3a f/+ ; Dnmt3b f/+ rep 1
GSM7157112 Sperm Dnmt3a f/+ ; Dnmt3b f/+ rep 2
GSM7157113 Sperm Dnmt3a f/- ; Dnmt3b f/- ; Stra8-iCre tg rep 1
GSM7157114 Sperm Dnmt3a f/- ; Dnmt3b f/- ; Stra8-iCre tg rep 2

Bulk RNAseq
GSM7157166 Undifferentiated Spermatogonia Dnmt3a f/+ ; Dnmt3b f/+ rep 1
GSM7157167 Undifferentiated Spermatogonia Dnmt3a f/+ ; Dnmt3b f/+ rep 2
GSM7157168 Undifferentiated Spermatogonia Dnmt3a f/+ ; Dnmt3b f/+ rep 3
GSM7157169 Undifferentiated Spermatogonia Dnmt3a f/- ; Dnmt3b f/- ; Stra8-iCre tg rep 1
GSM7157170 Undifferentiated Spermatogonia Dnmt3a f/- ; Dnmt3b f/- ; Stra8-iCre tg rep 2
GSM7157171 Undifferentiated Spermatogonia Dnmt3a f/- ; Dnmt3b f/- ; Stra8-iCre tg rep 3
GSM7157172 Differentiated Spermatogonia Dnmt3a f/+ ; Dnmt3b f/+ rep 1
GSM7157173 Differentiated Spermatogonia Dnmt3a f/+ ; Dnmt3b f/+ rep 2
GSM7157174 Differentiated Spermatogonia Dnmt3a f/+ ; Dnmt3b f/+ rep 3
GSM7157175 Differentiated Spermatogonia Dnmt3a f/- ; Dnmt3b f/- ; Stra8-iCre tg rep 1
GSM7157176 Differentiated Spermatogonia Dnmt3a f/- ; Dnmt3b f/- ; Stra8-iCre tg rep 2
GSM7157177 Differentiated Spermatogonia Dnmt3a f/- ; Dnmt3b f/- ; Stra8-iCre tg rep 3

SMARTseq2
GSM7157459 Early 4 cell stage embryo Dnmt3a f/+ ; Dnmt3b f/+ sperm derived rep 1
GSM7157460 Early 4 cell stage embryo Dnmt3a f/+ ; Dnmt3b f/+ sperm derived rep 2
GSM7157461 Early 4 cell stage embryo Dnmt3a f/+ ; Dnmt3b f/+ sperm derived rep 3
GSM7157462 Early 4 cell stage embryo Dnmt3a f/+ ; Dnmt3b f/+ sperm derived rep 4
GSM7157463 Early 4 cell stage embryo Dnmt3a f/+ ; Dnmt3b f/+ sperm derived rep 5
GSM7157464 Early 4 cell stage embryo Dnmt3a f/+ ; Dnmt3b f/+ sperm derived rep 6
GSM7157465 Early 4 cell stage embryo Dnmt3a f/+ ; Dnmt3b f/+ sperm derived rep 7
GSM7157466 Early 4 cell stage embryo Dnmt3a f/+ ; Dnmt3b f/+ sperm derived rep 8
GSM7157467 Early 4 cell stage embryo Dnmt3a f/+ ; Dnmt3b f/+ sperm derived rep 9
GSM7157468 Early 4 cell stage embryo Dnmt3a f/+ ; Dnmt3b f/+ sperm derived rep 10
GSM7157469 Early 4 cell stage embryo Dnmt3a f/+ ; Dnmt3b f/+ sperm derived rep 11
GSM7157470 Early 4 cell stage embryo Dnmt3a f/+ ; Dnmt3b f/+ sperm derived rep 12
GSM7157471 Early 4 cell stage embryo Dnmt3a f/+ ; Dnmt3b f/+ sperm derived rep 13
GSM7157472 Early 4 cell stage embryo Dnmt3a f/+ ; Dnmt3b f/+ sperm derived rep 14
GSM7157473 Early 4 cell stage embryo Dnmt3a f/+ ; Dnmt3b f/+ sperm derived rep 15
GSM7157474 Early 4 cell stage embryo Dnmt3a f/+ ; Dnmt3b f/+ sperm derived rep 16
GSM7157475 Early 4 cell stage embryo Dnmt3a f/+ ; Dnmt3b f/+ sperm derived rep 17
GSM7157476 Early 4 cell stage embryo Dnmt3a f/+ ; Dnmt3b f/+ sperm derived rep 18
GSM7157477 Early 4 cell stage embryo Dnmt3a f/+ ; Dnmt3b f/+ sperm derived rep 19
GSM7157478 Early 4 cell stage embryo Dnmt3a f/+ ; Dnmt3b f/+ sperm derived rep 20
GSM7157479 Early 4 cell stage embryo Dnmt3a f/+ ; Dnmt3b f/+ sperm derived rep 21
GSM7157480 Early 4 cell stage embryo Dnmt3a f/+ ; Dnmt3b f/+ sperm derived rep 22
GSM7157481 Early 4 cell stage embryo Dnmt3a f/- ; Dnmt3b f/- ; Stra8-iCre tg sperm derived rep 1
GSM7157482 Early 4 cell stage embryo Dnmt3a f/- ; Dnmt3b f/- ; Stra8-iCre tg sperm derived rep 2
GSM7157483 Early 4 cell stage embryo Dnmt3a f/- ; Dnmt3b f/- ; Stra8-iCre tg sperm derived rep 3
GSM7157484 Early 4 cell stage embryo Dnmt3a f/- ; Dnmt3b f/- ; Stra8-iCre tg sperm derived rep 4
GSM7157485 Early 4 cell stage embryo Dnmt3a f/- ; Dnmt3b f/- ; Stra8-iCre tg sperm derived rep 5
GSM7157486 Early 4 cell stage embryo Dnmt3a f/- ; Dnmt3b f/- ; Stra8-iCre tg sperm derived rep 6
GSM7157487 Early 4 cell stage embryo Dnmt3a f/- ; Dnmt3b f/- ; Stra8-iCre tg sperm derived rep 7
GSM7157488 Early 4 cell stage embryo Dnmt3a f/- ; Dnmt3b f/- ; Stra8-iCre tg sperm derived rep 8
GSM7157489 Early 4 cell stage embryo Dnmt3a f/- ; Dnmt3b f/- ; Stra8-iCre tg sperm derived rep 9
GSM7157490 Early 4 cell stage embryo Dnmt3a f/- ; Dnmt3b f/- ; Stra8-iCre tg sperm derived rep 10
GSM7157491 Early 4 cell stage embryo Dnmt3a f/- ; Dnmt3b f/- ; Stra8-iCre tg sperm derived rep 11
GSM7157492 Early 4 cell stage embryo Dnmt3a f/- ; Dnmt3b f/- ; Stra8-iCre tg sperm derived rep 12
GSM7157493 Early 4 cell stage embryo Dnmt3a f/- ; Dnmt3b f/- ; Stra8-iCre tg sperm derived rep 13
GSM7157494 Early 4 cell stage embryo Dnmt3a f/- ; Dnmt3b f/- ; Stra8-iCre tg sperm derived rep 14
GSM7157495 Early 4 cell stage embryo Dnmt3a f/- ; Dnmt3b f/- ; Stra8-iCre tg sperm derived rep 15
GSM7157496 Early 4 cell stage embryo Dnmt3a f/- ; Dnmt3b f/- ; Stra8-iCre tg sperm derived rep 16
GSM7157497 Early 4 cell stage embryo Dnmt3a f/- ; Dnmt3b f/- ; Stra8-iCre tg sperm derived rep 17
GSM7157498 Early 4 cell stage embryo Dnmt3a f/- ; Dnmt3b f/- ; Stra8-iCre tg sperm derived rep 18
GSM7157499 Early 4 cell stage embryo Dnmt3a f/- ; Dnmt3b f/- ; Stra8-iCre tg sperm derived rep 19
GSM7157500 Early 4 cell stage embryo Dnmt3a f/- ; Dnmt3b f/- ; Stra8-iCre tg sperm derived rep 20

RRBS
GSM7157523 Undifferentiated Spermatogonia Dnmt3a f/+ ; Dnmt3b f/+ rep 1
GSM7157524 Undifferentiated Spermatogonia Dnmt3a f/+ ; Dnmt3b f/+ rep 2

GSM7157525 Undifferentiated Spermatogonia Dnmt3a f/- ; Dnmt3b f/- ; Stra8-iCre tg rep 1
GSM7157526 Undifferentiated Spermatogonia Dnmt3a f/- ; Dnmt3b f/- ; Stra8-iCre tg rep 2
GSM7157527 Undifferentiated Spermatogonia Dnmt3a f/- ; Dnmt3b f/+ ; Stra8-iCre tg rep 1
GSM7157528 Undifferentiated Spermatogonia Dnmt3a f/- ; Dnmt3b f/+ ; Stra8-iCre tg rep 2
GSM7157529 Undifferentiated Spermatogonia Dnmt3a f/+ ; Dnmt3b f/- ; Stra8-iCre tg rep 1
GSM7157530 Undifferentiated Spermatogonia Dnmt3a f/+ ; Dnmt3b f/- ; Stra8-iCre tg rep 2
GSM7157531 Differentiated Spermatogonia Dnmt3a f/+ ; Dnmt3b f/+ rep 1
GSM7157532 Differentiated Spermatogonia Dnmt3a f/+ ; Dnmt3b f/+ rep 2
GSM7157533 Differentiated Spermatogonia Dnmt3a f/- ; Dnmt3b f/- ; Stra8-iCre tg rep 1
GSM7157534 Differentiated Spermatogonia Dnmt3a f/- ; Dnmt3b f/- ; Stra8-iCre tg rep 2
GSM7157535 Differentiated Spermatogonia Dnmt3a f/- ; Dnmt3b f/+ ; Stra8-iCre tg rep 1
GSM7157536 Differentiated Spermatogonia Dnmt3a f/- ; Dnmt3b f/+ ; Stra8-iCre tg rep 2
GSM7157537 Differentiated Spermatogonia Dnmt3a f/+ ; Dnmt3b f/- ; Stra8-iCre tg rep 1
GSM7157538 Differentiated Spermatogonia Dnmt3a f/+ ; Dnmt3b f/- ; Stra8-iCre tg rep 2
GSM7157539 Sperm Dnmt3a f/+ ; Dnmt3b f/+ rep 1
GSM7157540 Sperm Dnmt3a f/+ ; Dnmt3b f/+ rep 2
GSM7157541 Sperm Dnmt3a f/- ; Dnmt3b f/- ; Stra8-iCre tg rep 1
GSM7157542 Sperm Dnmt3a f/- ; Dnmt3b f/- ; Stra8-iCre tg rep 2
GSM7157543 Sperm Dnmt3a f/- ; Dnmt3b f/+ ; Stra8-iCre tg rep 1
GSM7157544 Sperm Dnmt3a f/- ; Dnmt3b f/+ ; Stra8-iCre tg rep 2
GSM7157545 Sperm Dnmt3a f/+ ; Dnmt3b f/- ; Stra8-iCre tg rep 1
GSM7157546 Sperm Dnmt3a f/+ ; Dnmt3b f/- ; Stra8-iCre tg rep 2

ChIPseq
GSM7157576 Sperm H3K4me3 Dnmt3a f/+ ; Dnmt3b f/+ rep 1
GSM7157577 Sperm H3K4me3 Dnmt3a f/+ ; Dnmt3b f/+ rep 2
GSM7157578 Sperm H3K4me3 Dnmt3a f/- ; Dnmt3b f/- ; Stra8-iCre tg rep 1
GSM7157579 Sperm H3K4me3 Dnmt3a f/- ; Dnmt3b f/- ; Stra8-iCre tg rep 2
GSM7157580 Sperm Nucleosome Dnmt3a f/+ ; Dnmt3b f/+ rep 1
GSM7157581 Sperm Nucleosome Dnmt3a f/+ ; Dnmt3b f/+ rep 2
GSM7157582 Sperm Nucleosome Dnmt3a f/- ; Dnmt3b f/- ; Stra8-iCre tg rep 1
GSM7157583 Sperm Nucleosome Dnmt3a f/- ; Dnmt3b f/- ; Stra8-iCre tg rep 2
GSM7157584 Sperm H3.3 Dnmt3a f/+ ; Dnmt3b f/+ rep 1
GSM7157585 Sperm H3.3 Dnmt3a f/+ ; Dnmt3b f/+ rep 2
GSM7157586 Sperm H3.3 Dnmt3a f/- ; Dnmt3b f/- ; Stra8-iCre tg rep 1
GSM7157587 Sperm H3.3 Dnmt3a f/- ; Dnmt3b f/- ; Stra8-iCre tg rep 2
GSM7157588 Sperm H3.1/2/t Dnmt3a f/+ ; Dnmt3b f/+ rep 1
GSM7157589 Sperm H3.1/2/t Dnmt3a f/+ ; Dnmt3b f/+ rep 2
GSM7157590 Sperm H3.1/2/t Dnmt3a f/- ; Dnmt3b f/- ; Stra8-iCre tg rep 1
GSM7157591 Sperm H3.1/2/t Dnmt3a f/- ; Dnmt3b f/- ; Stra8-iCre tg rep 2
GSM7157592 Undifferentiated Spermatogonia H3K4me3 Dnmt3a f/+ ; Dnmt3b f/+ rep 1
GSM7157593 Undifferentiated Spermatogonia H3K4me3 Dnmt3a f/+ ; Dnmt3b f/+ rep 2
GSM7157594 Undifferentiated Spermatogonia H3K4me3 Dnmt3a f/- ; Dnmt3b f/- ; Stra8-iCre tg rep 1
GSM7157595 Undifferentiated Spermatogonia H3K4me3 Dnmt3a f/- ; Dnmt3b f/- ; Stra8-iCre tg rep 2
GSM7157596 Differentiated Spermatogonia H3K4me3 Dnmt3a f/+ ; Dnmt3b f/+ rep 1
GSM7157597 Differentiated Spermatogonia H3K4me3 Dnmt3a f/+ ; Dnmt3b f/+ rep 2
GSM7157598 Differentiated Spermatogonia H3K4me3 Dnmt3a f/- ; Dnmt3b f/- ; Stra8-iCre tg rep 1
GSM7157599 Differentiated Spermatogonia H3K4me3 Dnmt3a f/- ; Dnmt3b f/- ; Stra8-iCre tg rep 2
GSM7157600 Undifferentiated Spermatogonia H3K36me3 Dnmt3a f/+ ; Dnmt3b f/+ rep 1
GSM7157601 Undifferentiated Spermatogonia H3K36me3 Dnmt3a f/+ ; Dnmt3b f/+ rep 2
GSM7157602 Undifferentiated Spermatogonia H3K36me3 Dnmt3a f/- ; Dnmt3b f/- ; Stra8-iCre tg rep 1
GSM7157603 Undifferentiated Spermatogonia H3K36me3 Dnmt3a f/- ; Dnmt3b f/- ; Stra8-iCre tg rep 2
GSM7157604 Differentiated Spermatogonia H3K36me3 Dnmt3a f/+ ; Dnmt3b f/+ rep 1
GSM7157605 Differentiated Spermatogonia H3K36me3 Dnmt3a f/+ ; Dnmt3b f/+ rep 2
GSM7157606 Differentiated Spermatogonia H3K36me3 Dnmt3a f/- ; Dnmt3b f/- ; Stra8-iCre tg rep 1
GSM7157607 Differentiated Spermatogonia H3K36me3 Dnmt3a f/- ; Dnmt3b f/- ; Stra8-iCre tg rep 2

Genome browser session
(e.g. UCSC)

https://genome.ucsc.edu/s/fanogrig/Fanourgakis%20et%20al%20Track%20Collection

Methodology

Replicates

There are 3 replicates for each biological group for ATATAseq and bulk RNAseq experiments. There are 2 replicates for each biological group for EMseq, RRBS and ChIPseq experiments. There are 20 and 22 replicates for the biological goups of the SMARTseq2 experiment.

Sequencing depth

For ATATAseq the mean yield per sample was 11,029,757 51-bp single-end reads, of which 8,861,903 reads were aligned to mm10 (80.34%).
For EMseq the mean yield per sample was 156,999,167 76-bp paired-end reads, of which 115,023,359 reads were aligned to mm10 (73.26%).
For bulk RNAseq the mean yield per sample was 31,783,548 38-bp paired-end reads, of which 22,661,358 reads were aligned to mm10 (71.29%).
For SMARTseq2 the mean yield per sample was 5,831,167 51-bp single-end reads, of which 4,578,847 reads were aligned to mm10 (78.52%).
For RRBS the mean yield per sample was 18,683,711 51-bp single-end reads, of which 13,414,841 reads were aligned to mm10 (71.79%).

For ChIPseq the mean yield per sample was 28,575,105 51-bp single-end reads, of which 23,069,955 reads were aligned to mm10 (80.73%).

Antibodies

anti-H3K4me3 (Millipore 17-614)
anti-H3K36me3 (Cell Signaling #4909)
anti-H3.3 (Cosmo Bio CE-040B)
anti-nucleosome provided by J. van der Vlag (Kramers, K., Stemmer, C., Monestier, M., van Bruggen, M.C., Rijke-Schilder, T.P., Hylkema, M.N., Smeenk, R.J., Muller, S. and Berden, J.H. (1996) Specificity of monoclonal anti-nucleosome auto-antibodies derived from lupus mice. J Autoimmun, 9, 723-729)
anti-H3.1/2/t provided by J. van der Vlag (van der Heijden, G.W., Dieker, J.W., Derijck, A.A., Muller, S., Berden, J.H., Braat, D.D., van der Vlag, J. and de Boer, P. (2005) Asymmetry in histone H3 variants and lysine methylation between paternal and maternal chromatin of the early mouse zygote. Mech Dev, 122, 1008-1022)

Peak calling parameters

ChIP-seq reads were first processed using TrimGalore (version 0.6.2) to trim adaptor and low-quality reads with settings (--stringency 3). Trimmed reads were then aligned to the mouse genome build mm10 using STAR (version 2.5.0a) with settings (--alignIntronMin 1 --alignIntronMax 1 --alignEndsType EndToEnd --alignMatesGapMax 1000 --outFilterMatchNminOverLread 0.85). No peak calling software was used. Peak calling was performed only for sperm anti-nucleosome and anti-H3K4me3 ChiPs using MACS3 with standard settings for broad peak search [macs3 callpeak --broad --nolambda -g mm --keep-dup all]. For downstream analysis we considered peaks with qvalue < 0.01, and lengths lower than the 99th quantie.

Data quality

We detected 45'125 and 50'354 anti-Nucleosome peaks, covering 1.2% and 0.8% of the genome, with a median length of 508 bp and 295 bp in Ctrl and DKO sperm, respectively (Supplementary Figure llB, llC, llD). We detected 26'654 and 29'166 anti-H3K4me3 peaks, covering 1.5% and 1.7% of the genome, with a median length of 950 bp and 953 bp in Ctrl and DKO sperm respectively (Supplementary Figure 12B, 12C, 12D).

Software

TrimGalore (version 0.6.2), STAR (version 2.5.0a), Bismark (version 0.23.3), GenomicFeatures (version 1.56.0), SAMtools (version 1.10), nudup.py (version 2.2), macs3 (version 3.0.0bl), Rstudio (R version 4.2.1), limma (version 3.52.2), QuasR (version 1.36.0), SCORPIUS (vl.0.8), PCAtools (version 2.8.0), stats (version 4.2.1), topGO (version 2.48.0), monalisa (version 1.10.1), edgeR (version 3.42.4)

Flow Cytometry

Plots

Confirm that:

☒ The axis labels state the marker and fluorochrome used (e.g. CD4-FITC).

☒ The axis scales are clearly visible. Include numbers along axes only for bottom left plot of group (a 'group' is an analysis of identical markers).

☒ All plots are contour plots with outliers or pseudocolor plots.

☒ A numerical value for number of cells or percentage (with statistics) is provided.

Methodology

Sample preparation

Testicular cell suspension was prepared by incubation of seminiferous tubules in 200 U/ml Collagenase type I (Worthington Biochemical LS004196), 5 µg/ml DNAse I (Roche 10104159001), and 0.05%Trypsin (Gibco 25200056) in GBSS (Sigma G9779) as described in (83). Cells were stained with 1/200 anti-CD117/c-kit PE (eBioscience 12-1171-83), anti-CD324/E-Cadherin eFluor 660 (eBioscience 50-3249-82) and anti-CD49f/Integrin alpha 6 PE-Cyanine7 (eBioscience 25-0459-82) for 1 hour at 32oC with constant shaking and protected from light. After washing cells were incubated with 20 µg/ml with Hoechst 33342 (Thermo Fischer Scientific H3570) for 1 hour at 32oC with constant shaking and protected from light and then with 30nM DRAQ7 (Biostatus DR71000) for 5 minutes on bench. The stained cells were strained through a 40µm nylon filter into 5 ml polypropylene tubes.
For sperm cell sorting, mouse cauda epididymides were dissected into a Petri dish and fat patches were removed with forceps and scissors. Each epididymis was punctured with a needle and carefully squeezed into 100 ul of PBS with the help of two forceps. Sperm was transferred into an Eppendorf tube and was allowed to liquefy at room temperature for 5 minutes. To break sperm tails, cells were briefly sonicated with a Brandson Tip digital sonicator with 10% amplitude and 3 cycles of 0.5 sec ON / 2 sec OFF. Cells were stained with 2 ul/ml Hoechst 33342 (H3570, ThermoFisher) for 1h at 25°C with constant shaking and protected from light. Sperm cells were filtered through a 40 µm Nylon filter into 5 ml polypropylene tubes.

Instrument

BD FACSAria III cell sorter (Becton Dickinson)

Software

BD FACSDiva (version 9), FlowJo (version 10)

Cell population abundance

Undifferentiated spermatogonia are 0.25% to 0.4% of the testicular suspension.
Differentiated spermatogonia are 0.75% to 1.2% of the testicular suspension.
Purity of the spermatogonia sorted populations was assessed by RNAseq expression of specific marker genes.
Purity of sperm cells was assessed by microscopy observation of the sorted cells.

Gating strategy

Cells were first gated for FSC and SSC to exclude debris. Then the live cells were selected based on the absence of DRAQ7 signal detected using 755LP, 780/60BP. Then we gated for cells positive for Hoechst 33342 emission which was detected using a 670LP (Hoechst-Red) and 450/20 BP (Hoechst-Blue). We gated for the Hoechst-Redlow and Hoechst-Bluemid population which is enriched for 2N spermatogonia. Fluorescence for PE was detected using a 582/15BP filter, for PE-Cy7 was

detected using 735LP,780/60BP filter and for eFluor660 using a 660/20BP filter. Undifferentiated spermatogonia were sorted as CD324high, CD49fhigh, CD117low and differentiated spermatogonia were sorted as CD324low, CD49flow, CD117high.

☒ Tick this box to confirm that a figure exemplifying the gating strategy is provided in the Supplementary Information.
